# Oral cavity hydrodynamics and drag production in Balaenid whale suspension feeding

**DOI:** 10.1371/journal.pone.0175220

**Published:** 2017-04-11

**Authors:** Jean Potvin, Alexander J. Werth

**Affiliations:** 1 Department of Physics, Saint Louis University, St. Louis, Missouri, United States of America; 2 Department of Biology, Hampden-Sydney College, Hampden-Sydney, Virginia, United States of America; University of Washington, UNITED STATES

## Abstract

Balaenid whales feed on large aggregates of small and slow-moving prey (predominantly copepods) through a filtration process enabled by baleen. These whales exhibit continuous filtration, namely, with the mouth kept partially opened and the baleen exposed to oncoming prey-laden waters while fluking. The process is an example of crossflow filtration (CFF) in which most of the particulates (prey) are separated from the substrate (water) without ever coming into contact with the filtering surface (baleen). This paper discusses the simulation of baleen filtration hydrodynamics based on a type of hydraulic circuit modeling commonly used in microfluidics, but adapted to the much higher Reynolds number flows typical of whale hydrodynamics. This so-called *Baleen Hydraulic Circuit* (BHC) model uses as input the basic characteristics of the flows moving through a section of baleen observed in a previous flume study by the authors. The model has low-spatial resolution but incorporates the effects of fluid viscosity, which doubles or more a whale’s total body drag in comparison to non-feeding travel. Modeling viscous friction is crucial here since exposing the baleen system to the open ocean ends up tripling a whale’s total wetted surface area. Among other findings, the BHC shows how CFF is enhanced by a large filtration surface and hence large body size; how it is carried out via the establishment of rapid anteroposterior flows transporting most of the prey-water slurry towards the oropharyngeal wall; how slower intra-baleen flows manage to transfer most of the substrate out of the mouth, all the while contributing only a fraction to overall oral cavity drag; and how these anteroposterior and intra-baleen flows lose speed as they approach the oropharyngeal wall.

## Introduction

**Fig 1:**
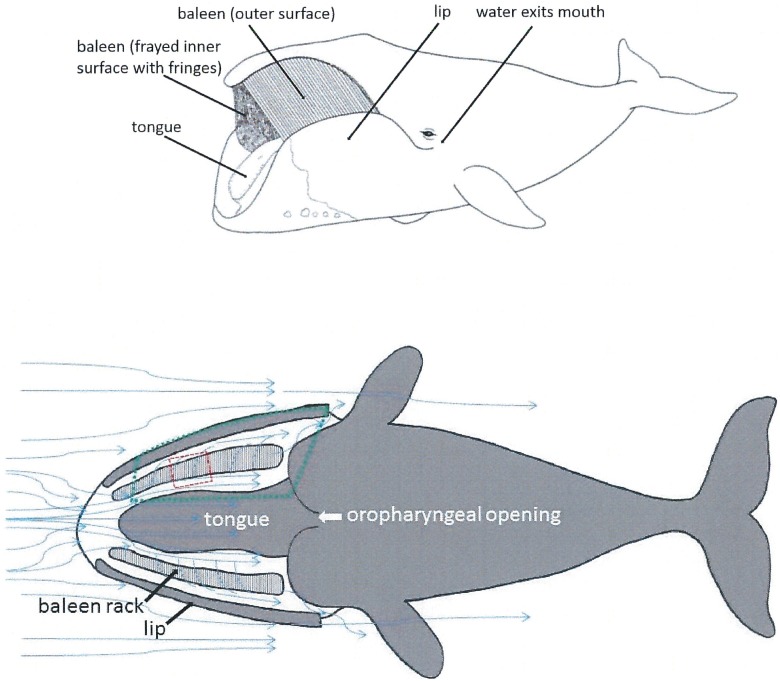
External and schematic (dorsal cutaway) views showing position of paired racks of 300 serial baleen plates between tongue and lips. In dorsal cutaway view with oral roof removed (bottom of figure), blue arrows indicate direction of water flow though and around baleen filtering apparatus in life as well as in experimental flow tank trials and computational modeling calculations (hypothetical but predicted from data of current study and previously published experiments [3, 5]). Water can flow anteroposteriorly (AP) within mouth along the tongue (APT channel) or the lip (APL channel). Filtered water exits the mouth via paired posterior openings (PO). Oropharyngeal opening which leads to esophagus lies near oral floor caudal to the tongue root. Dashed red box indicate the approximate location of the shortened mini-rack studied in a previous flow tank study [9]; dotted green box shows the system under consideration in this paper.

All members of the Suborder Mysticeti use baleen to separate the prey (particulates) from the sea water (substrate). However, the manners in which the filtration process takes place turn out to be quite different in each family, leading to different ways of generating drag. Balaenid whales, including the bowhead whale (*Balaena mysticetus* Linnaeus 1758) and right whales (*Eubalaena* spp. Linnaeus 1758), represent one of few groups of large-bodied organisms that use crossflow filtration (CFF) as a means to collect large amounts of small and slow-moving prey [1–9]. Baleen disposed longitudinally within the oral cavity is used to separate prey from the water, which flows continuously in and out of the mouth and filter due to the whales’ forward motion (Fig 1). The filtration is deemed as “crossflow” because most of the particulate moves parallel to the filtering surfaces (baleen) rather than into them as with “throughput” filtration [9, 10]. Here the prey directly accumulates at the oropharynx rather than being brought there by the tongue after sieving; in other words, a balaenid whale feeds on a suspension rather than a sieved mass tangled in the baleen and fringes after skimming [9]. This is crucial here, since the prey (primarily copepods) turn out to be significantly smaller in size than the width of the intra-baleen gaps forming the filtration surface [6, 10].

In contrast, and again during filtration, the baleen of rorquals (Balaenopteridae) are exposed not to the open ocean (except momentarily, during engulfment) but to the intraoral water-prey mixture engulfed during a lunge and contained within the closed buccal cavity [11–13]. And so the movement of water past and out through baleen is instead enabled by internal pressure buildup within the buccal cavity, as caused by the contraction of musculature embedded within the ventral groove blubber (VGB) [14, 15]. This intermittent filtration mechanism is also used by the gray whale, *Eschrichtius robustus*, although in gray whales the engulfment of a single mouthful of prey-laden water occurs via intraoral suction generation rather than by rapid lunges, and there are far fewer throat grooves to expand and hold engulfed water; relative to rorquals there is less intraoral pressure generation and less gular musculature to contract and expel the excurrent water past the baleen filter [1, 7, 16].

A quantitative description of these intra-oral flows for any of these mysticetes has remained elusive, however, due to the obvious impossibility of studying such large animals in the laboratory. Only by parsing the problem into parts amenable to experimental and theoretical investigation, and along with using kinematic data collected in the field, can we hope to further understand what is bound to remain out of view. Moreover, exhibiting what appears to be the least dynamic of the filtration strategies used by baleen whales, balaenid CFF offers the simplest flows available for study.

**Fig 2:**
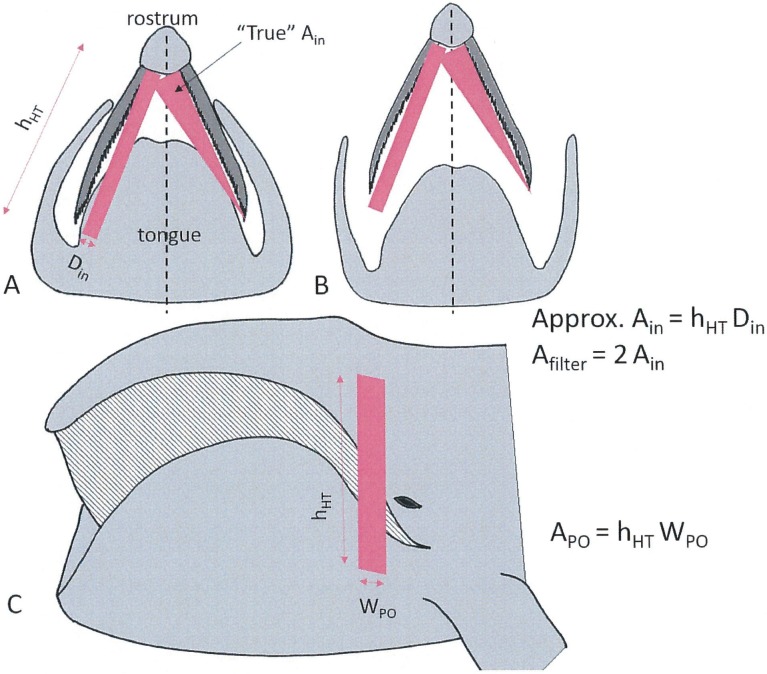
Schematic definitions of oral cavity inlet and outlet surface areas. Equivalent inlet area versus actual area, in two cases of lip emplacement and mandibular opening: semi-closed (A) and wide open (B). Definition of the flow exit area at the Posterior Opening (C).

As a first step towards this goal, a prequel paper [9] has discussed results of a flow tank study of baleen hydrodynamics near and through a section of real baleen, showing CFF to arise mainly from their cambered shape. This process is also enabled by the anteroposterior and mediolateral pressure gradients that split the current entering the mouth into a lateral intrabaleen (IB) flow and an anteroposterior (AP) flow moving towards the oropharyngeal wall (Fig 1). In the flow tank study the AP pressure gradient was created externally by the flume’s pump system, thereby recreating in a very approximate manner the lower pressure found near a whale’s Posterior Opening (PO). The AP flow is the faster of the two flows, thus allowing the transport of most of the engulfed slurry of prey towards the oropharynx. IB flow, whose sole purpose is to reduce the water contents of the slurry, is significantly slower but capable (simultaneously) of transferring a large mass of water, owing to the large surface area of the baleen racks in comparison with that of the mouth inlet (Figs 1 and 2; see also Fig 1 and 2c in [9]). The great efficiency of balaenid CFF stems from the generation of slow IB flows in conjunction with fast AP flows, which minimizes the number of prey items that pass through the filter or get entrapped in the baleen plates and fringes. Moreover, slow IB flows ensure that the time needed to clog the baleen and fringes (when happening) is much longer, in comparison with conventional “dead-end” sieving scenarios [9, 10]. Finally, the study shows the robustness of CFF in taking place regardless of the precise emplacement of the tongue and lip walls.

**Fig 3:**
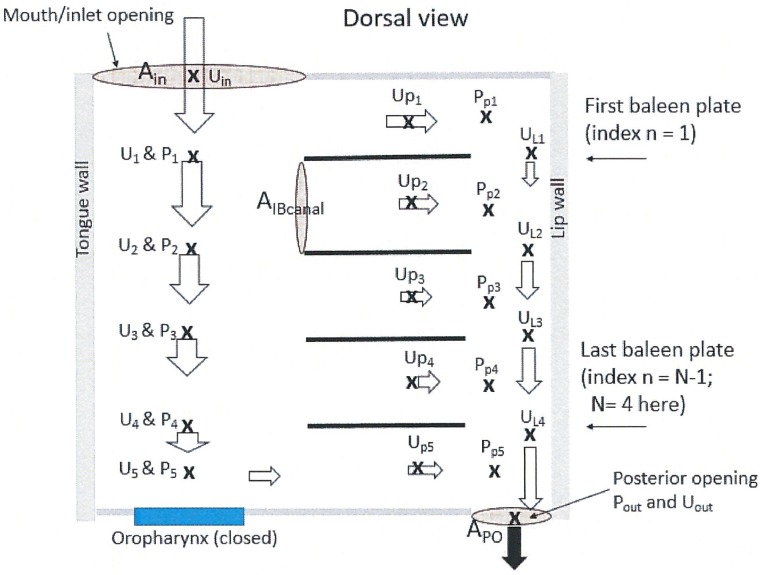
Schematic buccal cavity (right half, dorsal view) in BHC model of whale with four baleen plates and without flows into the oropharynx. Crosses mark locations of flow speeds (U) and pressures (P) calculated in the BHC model. Arrows represent the cross section-averaged flow speeds (the longer the arrow the faster). On the lingual side, anteroposterior flow decelerates because of the mass loss incurred by intra-baleen flow. On the labial side, flow accelerates from the mass gained from intra-baleen flows. Variables *A*_*in*_ and *A*_*po*_ correspond to surface areas of the gap between the baleen rack and tongue’s side wall and to the posterior opening (Fig 2).

Flow tank studies of balaenid baleen systems are by necessity limited in scope given the truly large sizes of these animals. With actual oral cavities measuring up to 3-5m in length (anteroposterior), 1-3m in height (dorsoventral), and 1-3m in width (mediolateral), the typical size of flumes (3m^3^ or less) makes hydrodynamic analysis of a fully-sized baleen rack all but impossible. Thus only small sections of baleen can be studied experimentally, to yield information with regards to the local flows near and through baleen [5, 9]. However, and thanks to a rack’s long serial axial symmetry where pressure and (flow) celerity gradients vary little over length scales of 0.3m or less (Fig 1), and where the camber and triangular shape of individual plates also vary little along the length of the whole baleen rack, such data can be used in mathematical modeling of the global flows characterizing the entire oral cavity. This has been done here via a hydraulic circuit approach commonly seen in microfluidics [17], but modified for the high Reynolds number flows relevant to balaenid hydrodynamics. A so-called *Baleen Hydraulic Circuit* (BHC) model was devised to calculate the pressure drops and flow speeds resulting from the viscous friction present in between the intra-baleen gaps and the body drag that ensues. This is a low-spatial resolution model, but simple enough to be solved by common spreadsheet programs. Its inputs includes morphological data characterizing the dimensions of the various canals defining the oral cavity (Figs 2 and 3), flume data to set the proportion of fluid moving into a particular baleen canal in relation to the mass that continues past it [9], and modeling of the viscous friction between the flows and oral tissue. These BHC calculations first yield an estimate of the extra drag force arising from baleen exposure to the open ocean, which in turn is used to infer oral cavity hydrodynamics. As shall be seen here, and in comparison to that of non-feeding travel, baleen exposure can *triple* the net wetted surface area of a whale, and increase total drag by 100% to 400% depending on mouth inlet area, or, in other words, on how much a whale lowers it mandibles and/or cant its lips (Fig 2). These calculations turn out to agree reasonably well with the drag inferred from the kinematic data collected with digital tags deployed on feeding whales in a glide [18].

Ultimately, knowing how much drag there is adds new insights in the trade-off between the energy gained through ingestion of prey and the energy lost through the metabolic expenditure of foraging. All of this has ecological implications, particularly with regard to the minimal copepod densities (~ 400/m^3^) and the not-to-exceed swim speed (~ 2m/s), needed to assure surplus harvest yields which on a yearly basis must guarantee survival over long fasting periods. These aspects and more shall be addressed in a sequel paper.

## Materials and methods

### The physical problem

Efficient filter design begins with packaging a large filtering surface area within an enclosure that generates the least drag [6, 9]. This is no different with balaenids, as exemplified by the case of a 10m, 15,155kg whale simulated here (Tables 1–4), for which longitudinally-oriented oral tissue exposes 144m^2^ of surface area to the open ocean. This is a large area in comparison to the 41.7m^2^ characterizing the external surface of its body (Fig 1 and Eq 4 below). These high values are due to the 126m^2^ wetted area of the two racks each comprising approximately 300 baleen plates [16] (Fig 1 and Table 3); and to long and tall labial and lingual walls each worth 9m^2^ of surface area. (The lingual wall area estimate attempts at including both surfaces of the tongue and oral cavity floor surface, albeit imperfectly). With such a large exposed filter comes the viscous friction generated by the boundary layers attached to these surfaces. This, in turn, significantly increases a whale’s drag, and to levels exceeding twice the drag sustained during non-feeding travel [18].

**Table 1:** BHC input data—Morphology.

Parameter	Body length 8m	Body length 10m	Body length 15m (Case A)	Body length 15m (Case B)	Comments
*N*_*b*_ = ***N-1* baleen count per rack**	299	299	299	299	
***d***_***baleen***_ **baleen separation (m)**	0.008	0.010, 0.015, 0.200	0.015	0.015	Also width of each IB canal
***c***_***baleen***_ **mean baleen chord (m)**	0.056	0.07	0.105	0.105	
***h***_***HT***_ **average depth of baleen under water (m)**	1.2	1.5	2.60	2.06	
***D***_***in***_ **width of the APT canal (m)**	0.4	0.30, 0.50, 0.72, 1.0	0.65	0.75	
***Total Body wetted area (m***^***2***^***) (closed mouth)***	24.0	41.7	93.5	84.0	Eqs 4 and 5

**Table 2:** BHC input data—Environmental characterization.

Parameter	Body size 8m	Body size 10m	Body size 15m (case 15A)	Body size 15m (case 15B)	Comments
**ρ**_**w**_ **density of sea water (kg/m**^**3**^**)**	1027	1027	1027	1027	
**υ kinetic viscosity (m**^**2**^**/s)**	1.46 x 10^−6^	1.46 x 10^−6^	1.46 x 10^−6^	1.46 x 10^−6^	Average over values ranging from 5°C and 10°C
**P**_**atm**_ **sea level atmospheric pressure (N/m**^**2**^**)**	101325	101325	101325	101325	
**Flow-splitting coefficient *C***	0.44	0.30, 0.40, 0.44, 0.50, 0.53	0.44	0.44	*a ≡ U*_*300*_*/U*_*in*_
**U**_**whale**_ **m/s**	0.8 to 4.0	0.8 to 15.0	0.8 to 4.0	0.8 to 4.0	
***Baleen “hydrofoil” drag coefficient***	0.25	0.25	0.25	0.25	See Modeling Details 5

**Table 3:** Hydrodynamic parameters of interest (10m whale; data from Tables 1 & 2). The flow speeds used here correspond to medium rack obstruction (C = 0.4). Flow speeds at lower obstruction (C > 0.4) would be higher.

Parameter		Comments
R_e_ APt canal(length-based)	3.1x10^6^ to 0.4x10^6^	U_i_ ranging from 1.5m/s (under the rostrum), down to 0.20m/s (near the oropharynx); length of canal = N_b_ d_baleen_ ~ 300 x 0.01m
R_e_ APt canal(diameter-based)	0.87x10^6^ to 0.11x10^6^	U_i_ ranging from 1.5m/s (under the rostrum), down to 0.20m/s (near the oropharynx); hydraulic diameter of canal = 2m = 2 N_b_ d_baleen_ D_in_/ (D_in_ N_baleen_ + d_baleen_)
R_e_ IB canal(length-based)	3.1x10^4^ to 2.4x10^3^	U_pi_ ranging from 0.65m/s (under the rostrum), down to 0.05m/s (near the oropharynx); length of canal = c_baleen_ ~ 0.07m
R_e_ IB canal(diameter-based)	0.89x10^4^ to 0.67x10^3^	U_pi_ ranging from 0.65m/s (under the rostrum), down to 0.05m/s (near the oropharynx); hydraulic diameter of canal = 0.02m = 2 c_baleen_ d_baleen_/ (c_baleen_ + d_baleen_)
Wetted area for all IB canals	126m^2^	= 2sides * 2racks * h_Ht_ c_baleen_ N_b_ c_baleen_ ~ 0.07m, N_b_ ~ 300, h_HT_ ~ 1.5m
Wetted area for APt canal	9m^2^	= 2sides * h_Ht_ d_baleen_ N_b_ Note: d_baleen_x N_b_ = baleen rack length;d_baleen_ ~ 0.01m, N_b_ ~ 300, h_HT_ ~ 1.5m
Wetted area for APl canal	9m^2^	2sides * h_Ht_ d_baleen_ N_b_
Boundary layer thickness at the end of IB canal near rostrum	3.2mm	Calculated via Eq 10; with U_pi_ ~ 0.65m/s and x = c_baleen_ ~ 0.07m
Boundary layer thickness at the end of IB canal near oropharynx	5.4mm	Calculated via Eq 10; with U_pi_ ~ 0.05m/s and x = c_baleen_ ~ 0.07m

**Table 4:** BHC input: Supplementary definitions and constraints.

**Definition**	*A*_*PO*_ = *w*_*PO*_ *h*_*HT*_	PO surface area
**Definition**	*A*_*in*_ = *D*_*in*_ *h*_*HT*_	Cross section area of the APT canal entrance (one side)
**Definition**	*A*_*IBchannel*_ = *d*_*baleen*_ *h*_*HT*_	Cross section area of the IB canal entrance
**Constraint**	*U*_*in*_ = *U*_*whale*_	As suggested from observations
**Constraint**	*P*_*PO*_ *< P*_*ext*_	See Modeling Details 2
**Constraint**	*U*_*out*_ *~ 1*.*15 U*_*whale*_	See Modeling Details 2
**Constraint**	*D*_*in*_ = *1*.*15 w*_*PO*_	See Modeling Details 2
**Constraint**	*U*_*ext*_ *~ 1*.*25 U*_*whale*_	See Modeling Details 2

The flow dynamics within the oral cavity takes place over a wide range of scales. First, fluids need to move over the entire length, height and width of the mouth, which span scales of one meter up to several meters as discussed previously. Water travels similar distances over the entire length (“span”) of baleen (1 to 2m), but also over much smaller distances along the few centimeters characterizing the width (or “chord”) of baleen (7cm), and through the gaps separating the baleen (1cm) [13]. With foraging swim speeds spanning a range of 0.3m/s to 1.2m/s [18, 19–22], and the generally decreasing internal longitudinal flow speeds towards the oropharynx [9], the Reynolds numbers associated with the latter stand in the range of *R*_*e*_ ~ 10^5^–10^6^ (Table 3). These contrast with intra-baleen flows where *R*_*e*_ ~ 4x10^3^ to *R*_*e*_ ~ 1x10^4^ owing to the much smaller pipe diameters involved (Table 3). (These values are at least twice as large as those characterizing the Hagen-Poiseuille pipe flows of many biological systems [23]). Yet smaller scales of 1mm or less are found in the boundary layer and turbulence present over and near the baleen plates, as well as over both tongue and lip walls.

Studying in the laboratory and numerically the effects of length scales spanning up to five orders of magnitude is difficult. Generally, Computational Fluid Dynamics (CFD) techniques are powerful and accurate enough to handle such a range. But these would require unaffordable costs in computer hardware and time with the problem at hand, since tripling the surface area exposed to flow would, in comparison to the already-challenging simulations of closed-mouth swimming [18, 24], require approximately five times more grid points. (In typical CFD simulations, the bulk of the computational domain’s grid points are found near and within the fluid’s boundary layer coating the exposed surfaces. Thus tripling the latter would in effect increase the associated volume by a factor ~ area^3/2^ ~ 3^3/2^). This is problematic given the substantially large input parameter space explored here (Tables 1 and 2). We note that attempts at using CFD to model balaenid filtration and drag have been described in [18]. Our approach has been to develop a simpler hydrodynamic simulation scheme, but one that is complete and fast enough to provide a first look into the relevant hydrodynamics, thereby providing a road map for future CFD and flow tank studies.

The BHC is a low spatial-resolution hydraulic circuit model capable of calculating the local pressures and flow speeds both anteroposteriorly and mediolaterally and through the baleen/fringe complex, while accounting for most of the viscous friction sustained by these flows. Note that using hydraulic circuit modeling excludes the existence of large scale vortical motions taking place over the largest scales of the mouth (i.e., ~ 1m), as has been invoked in other organisms [25]. Conceptually, this means that overall flow celerity will be driven by pressure gradients rather than inertial effects, as applied over the entire length, width and height of the mouth apparatus. Herein the main anteroposterior pressure gradient arises from the low pressure at the PO associated with the external flows accelerating around the oblong geometry of the body [3, 9]; and the medio-lateral pressure gradient arising across the racks due to baleen curvature [9].

The BHC model is described qualitatively in the following sections, with the detailed mathematics applied to the simpler and pedagogical 4-baleen system discussed in the six sections that follow the *Final Remarks* (*Modeling Details 1–6*). Application to the more general *N*_*b*_ ~ 300 baleen system is discussed in *Modeling Details 7*, which is followed by a list of the mathematical symbols and acronyms.

### Simulated oral apparatus

BHC modeling calculates the flows and pressures at the locations shown in Fig 3, including within each intra-baleen (IB) gaps, as well as in the anteroposterior gaps/canals, i.e., both lingual (APT) and labial (APL). Several sites are used for the calculation of both flow speed and pressure, while others are assigned to either quantity. The pressures *P*_*i*_ and *P*_*pi*_ (at site *i* along a rack) correspond to the pressures in the APT and APL canals respectively, and by extension to the pressure at both ends of the IB channel separating both AP canals there. Obviously, the rectangular cavity of Fig 3 is a rough approximation, in comparison to the actual, curved 3-dimensional cavity exemplified in Figs 1 and 2. This applies to the baleen system as well, where racks of straight, equal-span baleen is simulated as an approximation of the actual racks of curved and unequally-spanned baleen [16]. Although leading to a drastically simplified description of the fluid dynamics, the model cavity is based on the averaged dimensions found *in vivo*, and should yield useful estimates without losing too many of the important details. Note that in contrast to the simple system of Fig 3, the tongue of a whale does not present a boundary to the flow over the entire height of the mouth (Fig 2). Whether this is a good approach will depend on future studies of tongue emplacement and volume during feeding.

Another approximation involves calculating the viscous friction associated with an APL flow that origins entirely from the lateral motions (through baleen) of fluid initially located in the APT canal. This calculation omits altogether the contribution of the water that has entered the APL canal through the anterior opening of the lips (compare Figs 1 and 3). The effect that such additional flow has on overall APL flow and total body drag is currently unknown, and probably assessable only with more detailed CFD simulations. At present, it is suspected to be small, again due to the small of amount of extra fluid mass such opening allows in, compared to the mass flowing through the APT canal.

Finally, and given the assumed rectangular geometry of a BHC oral cavity, the resulting BHC solutions are equivalent to 3-dimensional flow moving horizontally in the bulk. In other words, the flow is the same dorsoventrally, in contrast to what may happen near a real section of baleen [9].

### BHC flow speed laws

**Fig 4:**
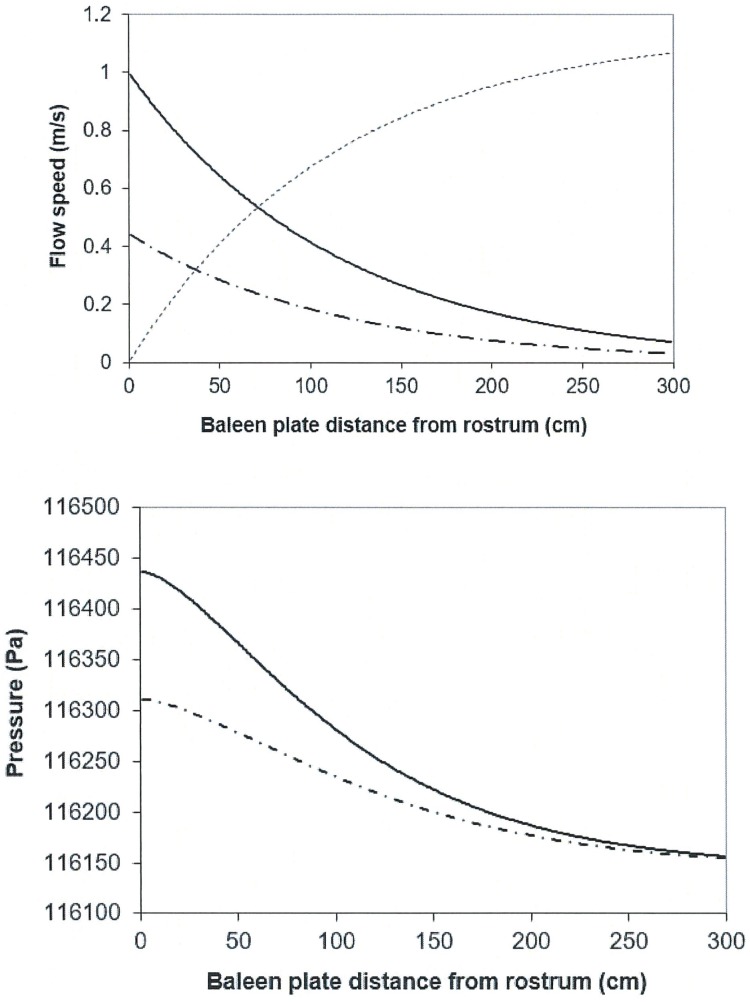
Calculated flow speeds and pressure drops for a 10m-whale swimming at U_whale_ = 1.0m/s. The IB, APT and APL flow speeds correspond to the dash-dot, continuous and dashed lines respectively; and the pressure profiles in the APT and APL canals likewise corresponding to the continuous and dash-dot lines. The inputs used in both frames are: 10m body length, d_baleen_ = 0.01m, c_baleen_ = 0.07m, h_HT_ = 1.5m and D_in_ = 0.5m, W_PO_ = 0.435m, a = 0.071 and C = 0.44 (“with prey” [9]). Further input details are listed in Tables 1 and 2.

The model enforces conservation of the flow mass rate entering and emerging from each canal (Fig 4) [9, 26]. It follows that the water entering the mouth and moving anteroposteriorly loses mass to the intra-baleen flow (Fig 1) and progressively decelerate as it approaches the oropharyngeal wall [9]. Such speed reduction occurs regardless of the existence of the pressure gradient arising from the high pressure at the mouth inlet and the low pressure at the posterior opening [3]. (In a single-outlet pipe, such a gradient would *accelerate* the flow). This trend of slowing APT flows has biological implications, including a slow-moving food slurry that is easier to swallow.

Calculation of the flow speeds are obtained after solving a system of equations implied by mass flow rate conservation. Details are shown in Tables 5 and 6, and Eqs 26–28 in *Modeling Details 7*. But complete solution isn’t achievable until a second set of equations connecting APT and IB flow speeds is defined. Used here is the *flow-splitting coefficient C*, which calculates the ratio of speeds within an intra-baleen canal (*U*_*IB*_) to the APT speed near the inlet of the given IB canal (*U*_*AP*_), or *C = U*_*IB*_ */U*_*AP*_. This coefficient can be measured in flume studies and is considered as constant in both time and space (i.e., over all IB canals). Values were extracted from data of the prequel paper [9], namely *C ~ 0*.*44 ± 0*.*04* and *0*.*53 ± 0*.*03*, obtained with and without the prey particles in the rack respectively. Interestingly, these results are independent of tongue wall locations (or APT canal width *D*_*in*_) and over the entire range of the swimming speeds used by foraging balaenids [14–17], an effect hypothesized to arise mostly from baleen camber [9]. Untested, however, is the dependence of *C* on the intra baleen separation (*d*_*baleen*_), thus limiting our sensitivity study to a narrow range (*d*_*baleen*_ = 0.01–0.02m). The ratio *U*_*IB*_ */U*_*AP*_ leads to the equations in the right column of Table 5, which after merging with those of the left column, yield the speed solutions shown in Table 6.

**Table 5:** Flow rate conservation (left) and flow splitting (right) equations in the 4-baleen BHC system. Indices correspond to the locations shown in Fig 3.

APT canal sections	IB canals
**$\begin{array}{l} A_{in}U_{in}=A_{in}U_{1}+A_{IBchannel}U_{p1}\\ A_{in}U_{1}=A_{in}U_{2}+A_{IBchannel}U_{p2}\\ A_{in}U_{2}=A_{in}U_{3}+A_{IBchannel}U_{p3}\\ A_{in}U_{3}=A_{in}U_{4}+A_{IBchannel}U_{p4}\\ A_{in}U_{4}=A_{in}U_{5} \end{array}$**	$\begin{array}{l} U_{p1}=CU_{in}\\ U_{p2}=CU_{1}\\ U_{p3}=CU_{2}\\ U_{p4}=CU_{3}\\ A_{baleen}U_{p5}=A_{in}U_{5} \end{array}$

**Table 6:** APT and IB flow solutions of the 4-baleen BHC system. Indices correspond to locations in Fig 3. A_IBchannel_ is the cross section area of the intra-baleen space and equal to the product h_Ht_ c_baleen_.

APT canal sections	IB canals
**$\begin{array}{l} U_{1}=U_{in}{\left(1-\frac{A_{IBchannel}}{A_{in}}C\right)}^{1}\\ U_{2}=U_{1}{\left(1-\frac{A_{IBchannel}}{A_{in}}C\right)}^{1}=U_{in}{\left(1-\frac{A_{IBchannel}}{A_{in}}C\right)}^{2}\\ U_{3}=U_{2}{\left(1-\frac{A_{IBchannel}}{A_{in}}C\right)}^{1}=U_{in}{\left(1-\frac{A_{IBchannel}}{A_{in}}C\right)}^{3}\\ U_{4}=U_{3}\left(1-\frac{A_{IBchannel}}{A_{in}}C\right)=U_{in}{\left(1-\frac{A_{IBchannel}}{A_{in}}C\right)}^{4}\\ U_{5}=U_{4} \end{array}$**	$\begin{array}{l} U_{p1}=U_{in}C\\ U_{p2}=U_{1}C=U_{in}{\left(1-\frac{A_{IBchannel}}{A_{in}}C\right)}^{1}C\\ U_{p3}=U_{2}C=U_{in}{\left(1-\frac{A_{IBchannel}}{A_{in}}C\right)}^{2}C\\ U_{p4}=U_{3}C=U_{in}{\left(1-\frac{A_{IBchannel}}{A_{in}}C\right)}^{3}C\\ U_{p5}=\frac{A_{in}}{A_{IBchannel}}U_{5}=\frac{A_{in}}{A_{IBchannel}}U_{4} \end{array}$

### BHC pressure drop laws; canal friction and boundary layer

The pressure differences at the ends of each canal are estimated with a type of pressure drop equations commonly used in constant-diameter pipe flow engineering [26], adapted here to the rectangular canal geometry implied in Fig 3. Being sustained in pipes/canals characterized by Reynolds numbers well-exceeding *Re* ~ 2000, the pressure gradient (*ΔP*) across both ends of a given canal is connected to the square of the flow speed at its inlet, i.e., *ΔP = k*_*canal*_ *½ ρ U*_*inlet*_^*2*^. Sometimes known as the Darcy–Weisbach equation, this relationship differs from the well-known Hagen-Poiseuille law where *ΔP* is only proportional to *U*_*inlet*_ [23]. Application of this formula is shown in Table 7 and this is where the BHC accounts for the viscosity and turbulence effects affecting the drag. Parts of the canal friction coefficient *k*_*canal*_, which varies at each canal, is calculated via the modeling of the flows as undeveloped flows, where the maximum lateral extent of the boundary layer coating the canal (flat) walls remain smaller than a canal half-width [26]. Moreover, and as further explained in *Modeling Details 4*, the coefficient is further adjusted to insure the absence of adverse pressure gradients within the cavity, along with pressure levels at the PO that are consistent with those exterior to the body.

**Table 7:** Pressure drop equation in the APT (left) and IB (right) canals in the simplified 4-baleen BHC system. Indices correspond to the locations shown in Fig 3.

APT canal sections	IB canals
**$\begin{array}{l} P_{in}-P_{1}=k_{APT}(1)\;\frac{1}{2}\rho_{w}U_{1}{}^{2}\\ P_{1}-P_{2}=k_{APT}(2)\;\frac{1}{2}\rho_{w}U_{2}{}^{2}\\ P_{2}-P_{3}=k_{APT}(3)\;\frac{1}{2}\rho_{w}U_{3}{}^{2}\\ P_{3}-P_{4}=k_{APT}(4)\;\frac{1}{2}\rho_{w}U_{4}{}^{2} \end{array}$**	$\begin{array}{l} P_{1}-P_{p1}=k_{IB}(1)\;\frac{1}{2}\rho_{w}U_{p1}{}^{2}\\ P_{2}-P_{p2}=k_{IB}(2)\;\frac{1}{2}\rho_{w}U_{p2}{}^{2}\\ P_{3}-P_{p3}=k_{IB}(3)\;\frac{1}{2}\rho_{w}U_{p3}{}^{2}\\ P_{4}-P_{p4}=k_{IB}(4)\;\frac{1}{2}\rho_{w}U_{p4}{}^{2}\\ P_{5}=P_{p5} \end{array}$

### Viscous friction drag of the mouth, versus total drag

The drag produced by the oral cavity and body is an important physical characteristic that provides the means of estimating the metabolic expenditures connected with swimming and feeding [27]. But calculating the drag from BHC output also allows for checking its accuracy since total body drag can be inferred from digital tag data collected in the field [18].

Drag measures the resistance encountered by a body moving through a fluid [23, 26]. In steady motions the common sources of drag are friction drag and pressure drag, with the latter arising from the turbulence of the whale’s near-wake [28], and the former from the viscous friction experienced by the fluid moving past the solid boundaries of exposed skin and baleen. Total drag is thus calculated from the sum of the mouth viscous friction drag (*F*_*D*_^*mouth*^) associated with the flows passing through the oral cavity, and the friction and pressure drag combination created by the (external) flows moving around the body (*F*_*D*_^*body*^):

$$F_{D}{}^{total}=F_{D}{}^{mouth}+F_{D}{}^{body}$$ (1)

where

$$F_{D}{}^{body}=\gamma \frac{1}{2}\rho_{w}S_{wetted}\left[1-\frac{2A_{in}}{S_{wetted}}\right]C_{D}{}^{closed\;mouth}U_{whale}{}^{2}$$ (2)

and

FDmouth=2DinhHT{12ρw(Uwhale2−UPO2)+(Pin−PPO)+12ρwCDbaleencbaleenLmouthdbaleenDin(21+Uwhale/UPO)3[12(Din+wPO)Lmouth]3Uwhale2} (3)

Eq 1 is an approximation that neglects the interaction between the external flow and the internal flow emerging out of the PO. Eq 2 corresponds to the standard drag equation used to model the effects of water resistance during non-feeding travel [18, 29–32]. The factor in brackets is an approximation of the wetted area of the body, minus that of the oral cavity inlet area. Even with an open mouth, most of a whale’s body–including the lips—deflects fluid around its exterior (Fig 1). The only area to subtract is the body feature that does not deflect fluid, namely that of the oral cavity inlet (*2A*_*in*_) (Fig 2). Parameters *C*_*D*_^*closed mouth*^ and *S*_*wetted*_ correspond to the non-feeding travel drag coefficient and wetted area of the body respectively, both in closed mouth configuration. The former is set at 0.0035 (15m body length) and 0.0059 (8 and 10m) per the glide study of Nousek McGregor [18], and the latter calculated using a correlation discussed in [29]

$$S_{wetted}=0.08M_{c}{}^{0.65}$$ (4)

The factor *M*_*c*_ corresponds to a whale’s body mass (kg) and is estimated from a correlation established in [33]:

$$M_{c}=13.2L_{body}{}^{3.06}$$ (5)

Finally, the factor γ represents a swimming depth correction factor that takes into account the (body) drag increases incurred when swimming near the surface [29, 34]. Herein γ = 1, corresponding to foraging bouts taking place at depths exceeding three times a whale’s body’s maximum (vertical) width (as in [18]).

Eq 3 describes the mouth friction drag term arising from the viscous and turbulent energy dissipated within the oral cavity. The first term is proportional to the total energy density–i.e., kinetic (½ *ρ*_*w*_*U*^*2*^) plus potential (pressure)—of the flows entering the mouth through area *A*_*in*_ at speed *U*_*in*_, minus that of the flows exiting the mouth through the PO at speed *U*_*PO*_. The difference measures the dissipative losses, viscous friction and turbulent, incurred by the flows while moving in the anterio-posterior direction (see *Modeling Details 5* for details). The second term represents the energy dissipated when the APT flows are redirected into IB flows, moving through baleen, and then redirected into APL flows. These effects are modeled here by looking at a rack of baleen as one of closely-spaced, high-angle-of-attack hydrofoils of known drag characteristics (*C*_*D*_^*baleen*^ = *0*.*25* here). This second term is calculated during input data entry and is used in the final adjustment of the IB canal friction coefficients *k*_*IB*_. A detailed derivation is described in *Modeling Details 5*.

The last ingredients are the drag coefficients *C*_*D*_^*total*^ and *C*_*D*_^*mouth*^ corresponding to total body drag and mouth-generated drag obtained from Eqs 1 and 3 respectively:

$$F_{D}{}^{total}=\frac{1}{2}\rho_{w}S_{wetted}C_{D}{}^{total}U_{whale}{}^{2}$$ (6a)

$$F_{D}{}^{mouth}=\frac{1}{2}\rho_{w}S_{wetted}C_{D}{}^{mouth}U_{whale}{}^{2}$$ (6b)

Usually the wetted area of the entire oral cavity would be the preferred reference surface area for the calculation of *C*_*D*_^*mouth*^. However, using the same (closed-mouth) body reference surface area *S*_*wetted*_ for both drag coefficients will permit their direct comparison when assessing the importance of mouth drag in comparison to that the drag generated by the rest of the body.

### BHC input parameters and constraints

Input parameters and constraints for BHC model calculation involve most known factors relevant to balaenid suspension feeding (see Figs 2 and 3 and Tables 1 and 2):

- Basic fluid dynamics: sea water density (*ρ*_*w*_), kinematic viscosity (*ν*), ocean static pressure measured at the surface (*P*_*atm*_) and whale swimming speed (*U*_*whale*_);
- Morphology: mean baleen chord (*c*_*baleen*_, assuming consistent serial axial morphology of plate geometry and camber, as mentioned above), spacing along rack (*d*_*baleen*_), and total number of plates on the rack (*N-1*);
- Width (*D*_*in*_) of the APT canal between tongue wall and medial side of a baleen rack;
- Lateral width of posterior opening (*w*_*PO*_) characterizing spacing of the APL canal between lip and labial end of the rack (See Eq 9 below);
- The baleen plate span (*h*_*HT*_), averaged over the length of a rack, used for filtration during foraging;
- Flow splitting coefficient (*C*), as extracted from flume data [9];
- Coefficients *f*_*APT*_, *x*_*IB*_, *y*_*rostr*_ and *z*_*rostr*_, used in the friction coefficient profiles calculated via Eqs 13–16 (see *Modeling Details 4*). Their values are adjusted to: 1) produce internal pressures at the PO that are consistent with pressures immediately external to it; 2) yield internal pressure profiles free of adverse gradients; and 3) insure viscous energy dissipation in between the baleen plates similar to that of the hydrofoil model used in Eq 3. This adjustment is described in *Modeling Details 1* and 4.

Parameters *D*_*in*_, *w*_*PO*_ and *h*_*HT*_ characterize morphological attributes that can be evaluated from field observations (ranges stated in Table 2), and likely to vary with the size and density of a prey patch. The calculations that take place during input data entry are further explained in *Modeling Details 1*.

## Results

### Pressures and flow speeds

Fig 4 presents examples of the computed flow speeds and pressures in the case of a 10m whale swimming at 1m/s. Not shown are the values of the IB flows in between the last baleen and the oropharyngeal wall (*U*_*p5*_ in the 4-baleen case of Table 5) which are typically high, but erroneous given the lack of knowledge on the size and geometry of that area. The figure shows both APT and IB flows becoming slower further down the rack, a direct result of the APT flows losing mass to the IB canals per conservation of the mass rate [9]. Greatly reduced speeds near the oropharynx are desirable as they will facilitate the swallowing of the food slurry accumulating in that area. The labial AP flows (APL) are shown as well, and are calculated from Eqs 24 and 25 (*Modeling Details 7*). As expected, this flow increases in speed as it moves along the canal, after picking up the fluid mass exiting the intra-baleen channels. The figure shows a similar comparison with the pressure drops sustained. Along with expectations, the AP pressures are greater than the IB pressures over the entire length of the rack. Moreover, both monotonically decrease to similar values near the oropharyngeal wall. This pattern thus excludes backflows in any one of the canals located upstream, as they are inconsistent with the flow solutions of Table 6 and Eqs 27 and 28, and unlikely to occur in a real whale. Finally, each calculated pressure profile is continuous within numerical discretization variations, to yield the same pressure difference between any two points in the cavity (within a fraction of a percent), as calculated from integrating pressure increments along arbitrary paths in the APT and/or APL canals starting and ending at those same two points.

**Fig 5:**
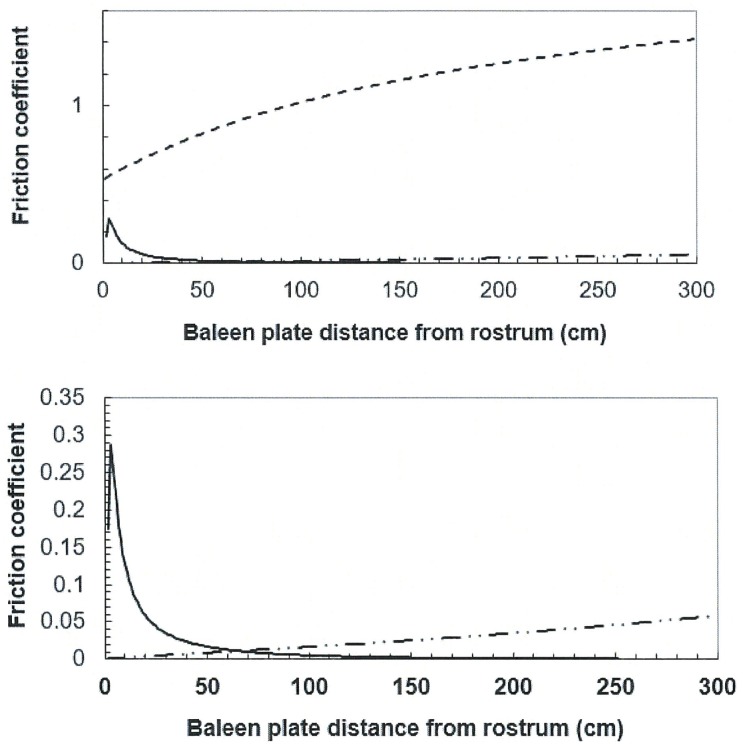
Canal friction coefficient for a 10m-whale swimming at U_whale_ = 1.0m/s. In the top frame the values of k_IB_, k_APL_ and k_APT_ are represented by the dashed, continuous and dot-dashed lines respectively. The values of the last two are shown again in the bottom frames. These are calculated with the same input data as in Fig 4. The figure shows a trend seen at all speeds, morphology and body size.

Typical values of the friction coefficients *k*_*canal*_ are shown in Fig 5 (calculated from Eqs 13–16). Generally, the coefficients are large where the boundary layer is “thick” (relative to pipe diameter) and/or when the flow speed is small. The values obtained for the IB flows are quite similar in order of magnitude to those encountered in microfluidic and hydraulic flows moving through bends of various angles [35, 36]. The friction coefficients of the wider APT and APL canals are significantly smaller in comparison but also comparable to the values encountered in similarly-sized straight- pipe and nozzle flows of similar Reynolds numbers [26, 36, 37].

**Fig 6:**
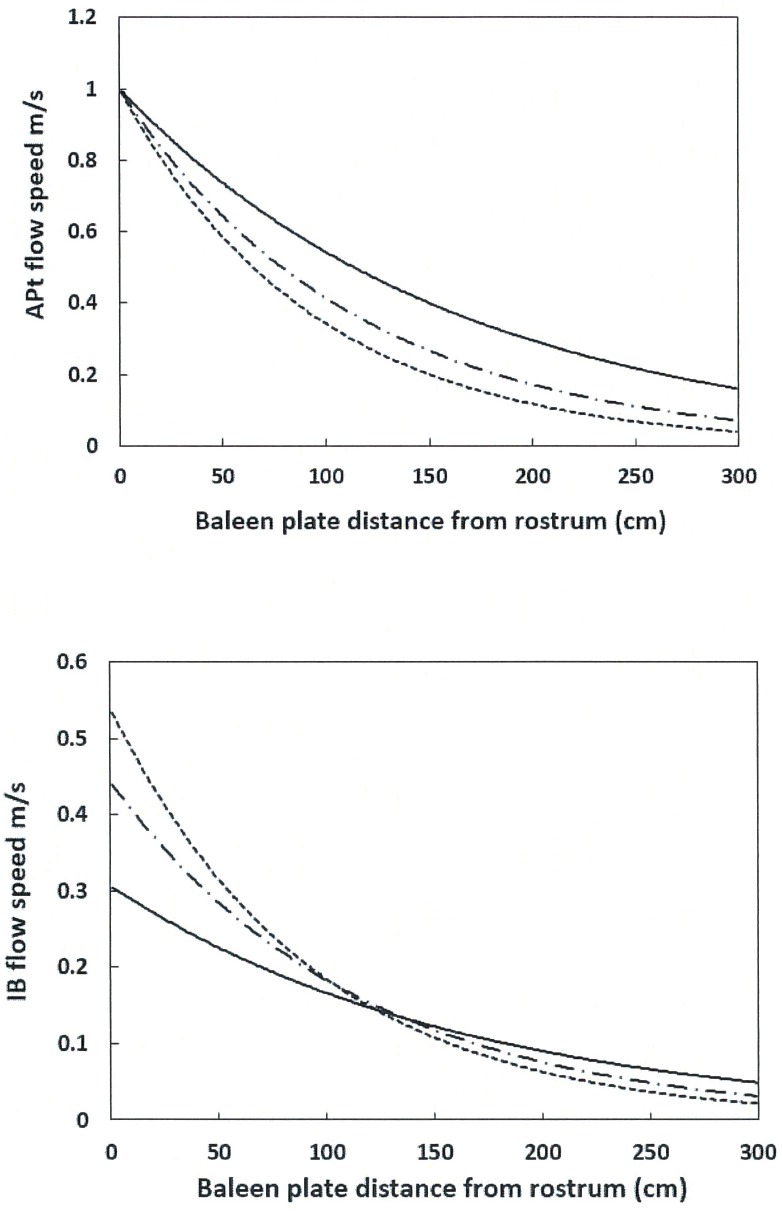
IB and APT flow speeds versus flow-splitting coefficient *C*. The larger the value of the coefficient, the larger the baleen obstruction by trapped prey. Shown are *C* = 0.30 (continuous curve), *C* = 0.44 (dash-dot; “with prey” [9]) and *C* = 0.53 (dotted; “no prey” [9]). All three cases involve the same mouth inlet flow rate. The rest of the inputs used in both frames where: 10m body length, *U*_*whale*_ = 1.0m/s, *d*_*baleen*_ = 0.01m, *c*_*baleen*_ = 0.07m, *h*_*HT*_ = 1.5m and *D*_*in*_ = 0.5m. Further details are listed in Tables 1 and 2.

Flow speeds obtained at different values of the flow-splitting coefficient *C* are shown in Fig 6. Different values of *C* correspond to various states of baleen canal obstruction by the prey tangled with the baleen fringe fibers [9]. Clean baleen would be characterized by *C ~ 0*.50, and *C ~ 0*.40 by obstruction levels likely to occur with particulate densities found in the field. Lower values are possible and presumably correspond to yet higher levels of rack obstruction.

The figure shows that the lower the *C*, the slower the IB flow in the anterior third section of the baleen rack, but faster in the posterior two-thirds. This pattern is necessary to keep up with the system’s in-flow mass rate which is held at the same value in the figure. In the APT canal, lowering *C* translates into faster speeds along the entire rack. To put it in another way, both flows would be concentrated along the anterior portion of the baleen rack at the higher values of *C*, but become more evenly distributed along the rack at lower values. Such a pattern suggests that baleen clogging would occur at first along the anterior section of the rack, to progressively expand further down the rack over time. This isn’t happening here, with the BHC being essentially a steady-state simulation of the flows. Another important aspect that has been left out of the figure is the decreasing mouth inlet flow rate *U*_*in*_ and higher inlet pressure *P*_*in*_ that would also accompany increased baleen clogging. Implementing this scenario over time would have resulted in the gradual lowering of the APT and IB flows of Fig 6 to levels lower than shown, ultimately to near-zero values.

**Fig 7:**
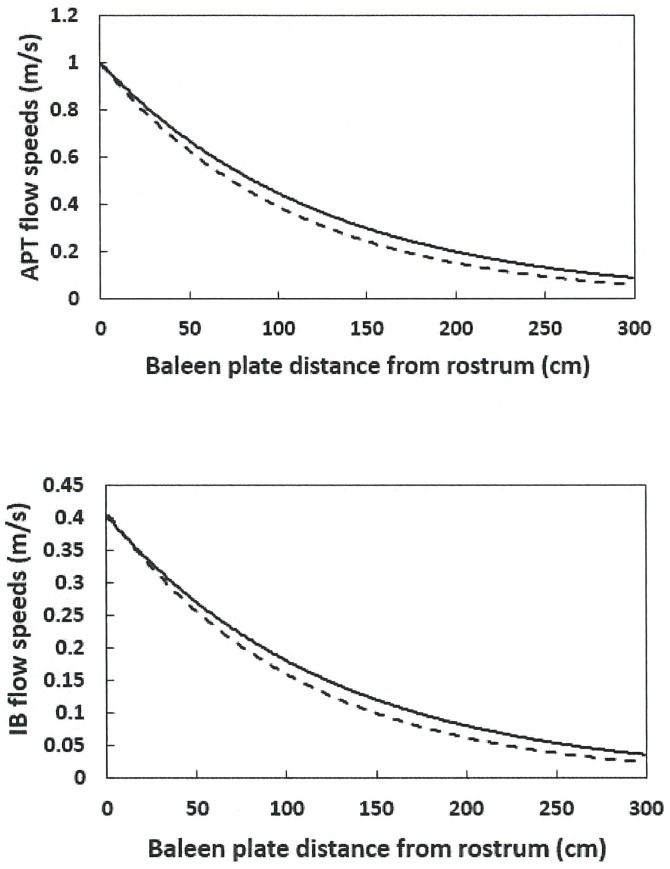
BHC flow speeds versus body size. Shown are values corresponding to body lengths of 10m (continuous line) and 15m –case A (dashed line). Both cases with simulated with *U*_*whale*_ = 1.0m/s and C = 0.40. At a 10m-length, d_baleen_ = 0.01m, c_baleen_ = 0.07m, h_HT_ = 1.5m and D_in_ = 0.5m; and at 15m, d_baleen_ = 0.015m, c_baleen_ = 0.105m, h_HT_ = 2.6m and D_in_ = 0.65m.

**Fig 8:**
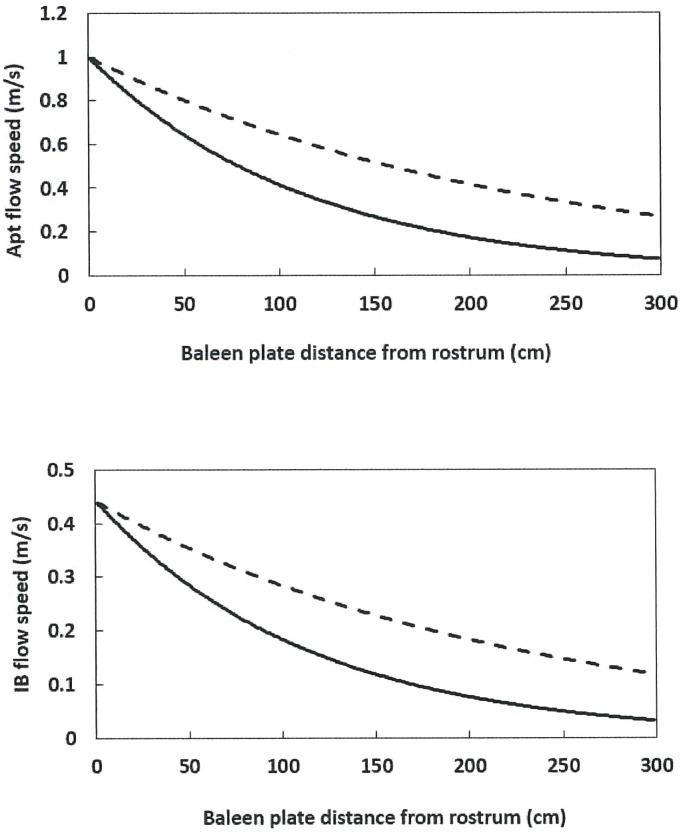
BHC flow speeds versus mean tongue wall distance from baleen (*D*_*in*_). Shown are values for widths of *D*_*in*_ = 0.5m (continuous line) and 1.00m (dashed line), in the case of a 10m whale swimming at *U*_*whale*_ = 1.0m/s. The rest of the inputs in both frames are as follows: *d*_*baleen*_ = 0.01m, *c*_*baleen*_ = 0.07m, *h*_*HT*_ = 1.5m and *C* = 0.44.

Figs 7 and 8 display how flow speeds change versus body size (i.e., 10m versus 15m), and tongue distance from the medial edge of the baleen rack (*D*_*in*_ = 0.5m versus 1.0m). Note that the comparison in Fig 7 is quite tentative since balaenid morphology versus body size isn’t well-known. The same could be said about the precise values of tongue wall emplacement. What the simulation shows, however, is that tongue emplacement is the more important factor, not only for flow speed, but also for drag generation as shown next.

### Mouth viscous friction drag

**Fig 9:**
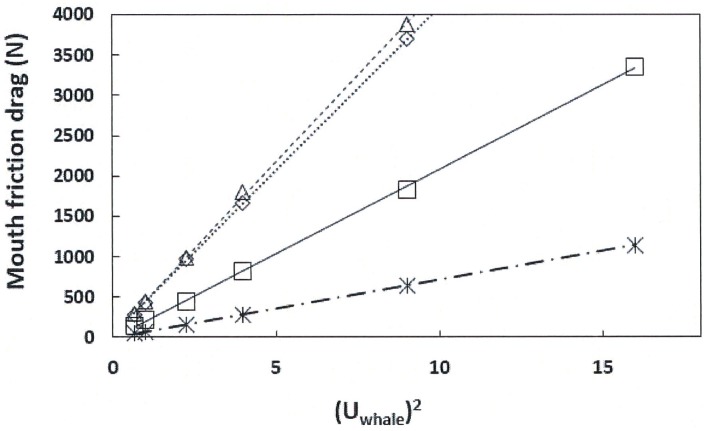
Calculated mouth friction drag versus the square of the swim speed and body size. Calculated via Eq 3 using the morphological inputs of Table 2 in the case *C = 0*.*44* (“with prey” [9]). (Note—with 10m, *d*_*baleen*_ was set at 0.01m). The body lengths considered were 8m (starburst and dash-dot line), 10m (squares and continuous line), 15m-case B (diamonds and dotted line) and 15m-case A (triangles and dashed line) respectively. The slopes corresponding to the lines are as follows: 71.26 kg/m (8m; R^2^ = 0.99), 207.95 kg/m (10m; R^2^ = 0.99), 407.79 kg/m (15m-case B; R^2^ = 0.99) and 431.98 kg/m (15m-case A; R^2^ = 0.99). The presence of the straight lines in this plot suggest a swim speed-independent but body size-dependent drag coefficient for the drag generated by the internal flows of the oral apparatus (Eq 6b).

**Fig 10:**
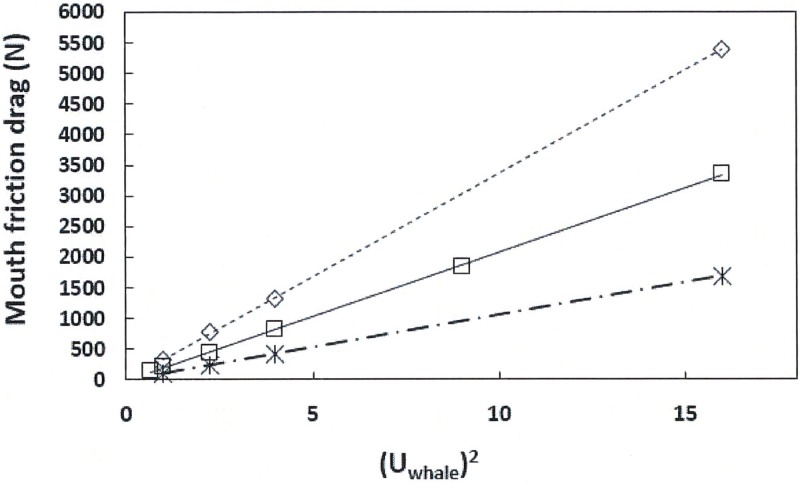
Mouth friction drag versus the square of the swim speed and mouth inlet parameters (*D*_*in*_). Calculated for 10m body length via Eq 3. Here *d*_*baleen*_ = 0.01m, *c*_*baleen*_ = 0.07m, *h*_*HT*_ = 1.5m, and *C = 0*.*44* (“with prey” [9]). The mouth inlet widths considered were *D*_*in*_ = 0.30m (starburst and dash-dot line), *D*_*in*_ = 0.50m (squares and continuous line) and *D*_*in*_ = 0.72m (diamonds and dotted line) respectively. The slopes corresponding to the lines are as follows: 105.57 kg/m (8m; R^2^ = 0.99), 207.95 kg/m (10m; R^2^ = 0.99), 337.04 kg/m (15m-case B; R^2^ = 0.99) and 431.98 kg/m (15m-case A; R^2^ = 0.99). As with Fig 9, the presence of the straight lines in this plot suggest a swim speed-independent but *D*_*in*_-dependent drag coefficient for the drag generated by the internal flows of the oral apparatus (see Eq 6b).

Values of the mouth friction drag calculated via Eq 3 are displayed in Figs 9 and 10, both versus the square of the swim speed. The former is a comparison among the three body sizes investigated, namely 8m (with a 2.4m-long oral cavity), 10m (3m-long) and 15m (4.5m-long). All three sizes feature different baleen spacing in the range of 0.01–0.02m (Table 1). Fig 10 shows the mouth drag generated by a 10m case in which three APT canal widths have been simulated (*D*_*in*_ = 0.30, 0.50 and 0.72m). All simulations were performed with 299 baleen plates and at value of the flow-splitting coefficient *C* equal to 0.44 (“with prey” [9]).

Comparing these figures show an unambiguous proportionality with *U*_*whale*_^*2*^ for all cases investigated, with the slopes strongly dependent on the value of *D*_*in*_ in Fig 10, and on body size in Fig 9. With respect to the latter, Eq 3 suggests that these variations in slope have more to do with the value of the inlet area *D*_*in*_ *h*_*HT*_ than with body size. Here the values of *D*_*in*_ *h*_*HT*_ turned out at 0.48m^2^ (8m body length), 0.75m^2^ (10m), and 1.69 and 1.54m^2^ (15m; case A and B respectively). In Fig 9 (10m body length) these inlet areas varied in a similar fashion, namely, at *D*_*in*_ *h*_*HT*_ = 0.45, 0.75 and 1.08m^2^. These results show that inlet cross section area is the dominant factor in oral cavity-generated drag across body sizes, with the implication that drag-control during foraging is to be mainly achieved by mandible and lip movement.

**Fig 11:**
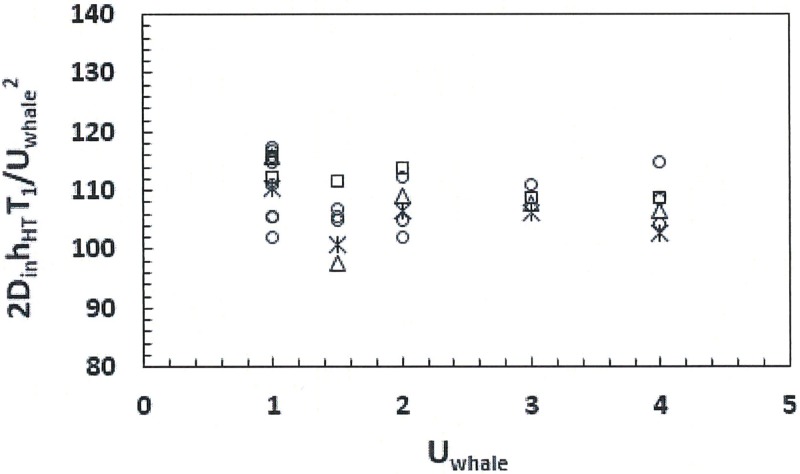
Scaling of the pressure plus kinetic energy difference between both ends of the oral cavity, versus swim speed. This is the first term in the curly brackets of Eq 3, also called *T*_*1*_ in the text, normalized into kg/m. Data at each body size are represented as follows: 8m (starbursts), 10m (circles), 15m-case A; (squares) and 15m-case B (triangles). Note that each body size may include cases with different values of *D*_*in*_ and *h*_*HT*_.

The slopes listed in the captions of Figs 9 and 10 should allow the interested reader to compute mouth drag at various speeds without ever running the BHC. But another, and more general correlation can be derived from the numerical data and Eq 3 as follows. Fig 11 shows the ratio of the first term “*T*_*1*_” within the curly brackets of Eq 3, here normalized as *2T*_*1*_*D*_*in*_*h*_*HT*_ /*U*_*whale*_^*2*^, at several values of swim speed and areas *D*_*in*_ *h*_*HT*_. “*T*_*1*_” is an estimate of the drag (per unit area) created by the viscous dissipation of the flows while moving longitudinally along both APT and APL canals (*Modeling Details 5*). It would appear that this ratio remains at about *2T*_*1*_*D*_*in*_*h*_*HT*_ /*U*_*whale*_^*2*^ *~* 109 kg/m (±10%) over all sizes and speeds considered. (This “10%” is associated with the variations that occur while tuning the *f*_*APT*_ coefficient). Using this value in Eq 3 permits complete reconstruction of the BHC-calculated mouth drag, i.e., with racks of 300 baleen each, and with the morphology not too far removed from Table 1. More importantly, it also permits the understanding of the scaling of mouth drag with respect to all of the relevant morphological inputs: for example, of *T*_*1*_ scaling linearly with *D*_*in*_, in contrast to the second term of Eq 3 (“*T*_*2*_”) scaling with the cube of *D*_*in*_; or with *T*_*1*_ being largely insensitive to baleen spacing (*d*_*baleen*_), versus *T*_*2*_ which is inversely proportional to *d*_*baleen*_^*3*^ (via *L*_*mouth*_ defined as *L*_*mouth*_ = *N*_*b*_ *d*_*baleen*_).

Most interestingly, such differential scaling ensures that about 60 to 90% of the mouth drag is generated by the *T*_*1*_-term, i.e., AP flows energy dissipation, with the *T*_*2*_–term representing that of the baleen and being important only in cases of large *D*_*in*_ (> 1m) used in conjunction with small *d*_*baleen*_ (< 0.01m). (In such cases *T*_*2*_ ~ 50% of the total mouth drag). Even though baleen exposed to seawater triples the wetted area of the body, it appears that their contribution to the drag turn out to be more modest. Per the BHC, this would come, mainly, from the significantly slower IB flows, in comparison to AP flows, as shown in Fig 4 (and also by Eq 18 in *Modeling Details 5*). The energy-dissipating fluid mass (per unit time) in both AP and IB canal systems are the same but the flow speeds being larger in the former insures that it becomes the most important sink of fluid energy. In retrospect, one wonders at how such lower contribution to mouth drag by baleen might have helped balaenids evolve larger filter sizes in the first place.

Another scaling property of Eq 3 is the mouth drag increasing linearly with the average length of baleen (*h*_*HT*_). This scaling is trivial here since both AP and IB flows are defined to move strictly in the horizontal (coronal) plane of a rectangular cuboidal cavity of uniform depth. On the other hand, scaling with respect to flow-splitting (*C*) appears minor in comparison, yielding 10% changes in mouth drag in the range *C ~* 0.3–0.5.

**Fig 12:**
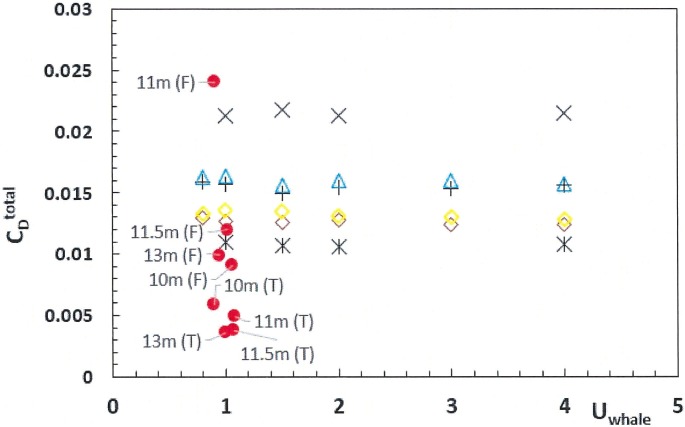
Coefficient of total (*C_D_^total^*) versus swim speed and body size. Calculated from Eqs 1–3 and 6a and for C = 0.44 (“with prey” [9]). Shown are the following body sizes: 8m (blue triangles); 10m (all black symbols, with *D*_*in*_ = 0.50m (crosses), *D*_*in*_ = 0.72m (times) and *D*_*in*_ = 0.30m (starbursts); and 15m (yellow diamonds for case A and brown diamonds for case B). This numerical data is compared with the drag coefficient calculated from kinematic data collected during feeding (red filled circles labeled “F”) and non-feeding transport (red filled circles labeled “T”) [18]. Note that the foraging swim speed of balaenids has been observed in the range of 0.6 to 2m/s [18, 19–22].

In comparison to the 100–165N total drag sustained during non-feeding travel at 1m/s [12], the viscous friction of the mouth turns out to be as large if not larger, as with the case of the 8m (*F*_*D*_^*mouth*^ 130N), 10m (211N) and 15m whales investigated here (444N with case A; 435N with case B). Thus mouth drag and mouth drag coefficient (*C*_*D*_^*mouth*^) end up doubling, tripling and even quadrupling total drag, depending of the amount of mouth inlet area. This trend is shown in Fig 12 which the coefficient of total drag *C*_*D*_^*total*^ (calculated from Eq 6a) versus swim speed. This data is also compared with those of a field study of foraging and traveling right whales [12]. Both sets of data (numerical vs. experimental) appear in agreement with respect to the drag-range that can be generated, presumably through the variation of mouth inlet area (*D*_*in*_ *h*_*HT*_).

### Energy dissipation pattern in the IB canals along the baleen rack

**Fig 13:**
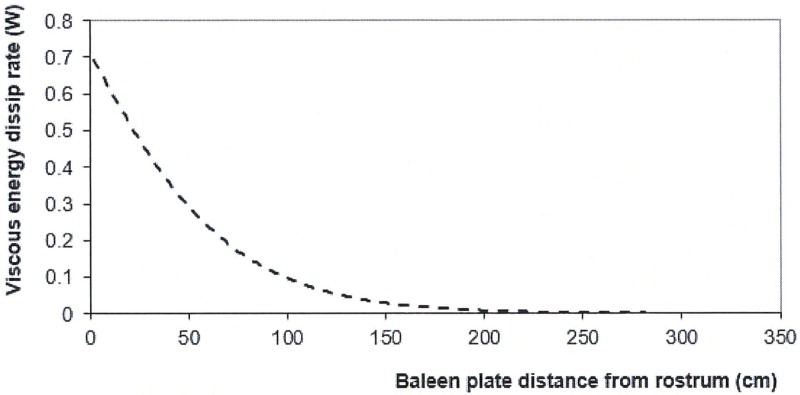
Viscous energy dissipation rate in each IB canals. Example of the 10m whale featured in Figs 4 and 5. Data summation yields the total energy dissipated by the baleen system and yields a contribution to mouth drag (Eq 20) identical to the hydrofoil model of Eq 3.

A rack of baleen can be seen not only as an assemblage of hydrofoils, but also as a parallel network of canals each independently dissipating viscous energy. From the latter perspective emerges an alternate approach to estimating energy dissipation and attendant drag codified in Eq 20 below. As further discussed in *Modeling Details 5*, each canal (located in between baleen plates *i* and *i+1*) dissipates viscous energy at a rate given by *D*_*in*_*h*_*HT*_*U*_*pi*_ *x k*_*IB*_*(i) x ½ ρ*_*w*_*U*_*pi*_^*2*^. Such a rate (at each baleen station) is shown in Fig 13 and turns out to be maximal along the anterior third of each rack. Although more difficult to quantify, the canal sections that forms the APT canal would show an analogous pattern, if only for the reason that APL flows are the fastest there as well (Fig 4).

## Discussion

### Prey collection and drag control

The hydrodynamic modeling described here has highlighted two novel aspects of balaenid cross-flow filtration hydrodynamics, namely the pattern of decelerating APT and IB flows towards the oropharyngeal wall; and the substantial increase of body drag, as contributed by the mouth’s viscous friction by flows going past the lingual and labial walls as well as through a very large number of tall and narrow baleen canals. The connection between these is key to understanding how the collection rate of prey, as determined by the rate of seawater intake *D*_*in*_*h*_*HT*_*U*_*whale*_, can be balanced against the generation of drag and attendant metabolic expenditures in the energy management of foraging. Thus in-flow rate is adjusted by the separate or combined use of the swim speed and mouth opening area, the latter achieved via the lowering of the mandibles along with the canting of the lip walls (Fig 2). All three parameters are also major determinant of drag (Eq 3 and Figs 9, 10 and 12), but in different proportions. Doubling the swim speed to collect twice as many prey items (per unit time) would lead to quadrupling the drag and required fluking thrust. As further explored in a sequel paper, there will be a speed “of diminishing returns” beyond which the collected prey will not yield enough energy to compensate for the metabolic activity of harvest. Interestingly, BHC modeling suggests that increasing mouth area instead of increasing the swim speed is the better strategy, as both drag and in-flow rate are both proportional to mouth area. The narrow range of swim speeds observed in the field, namely 0.6–1.2m/s [18, 19–22] would certainly be consistent with this hypothesis.

### Comparative biomechanics of drag production

Balaenids and rorquals differ in how they create drag during filtration. With the former, drag is caused mainly by the viscous friction sustained by water moving anterioposteriorly past the lingual and labial walls, and to a smaller extent, through the baleen canals. Exposing about 600 baleen plates to ocean flows ends up tripling the body wetted area and double or triple the drag sustained with a closed mouth (Fig 12) (See also [18]). With rorquals, body drag during filtration is caused entirely by the friction and pressure drag generated over the tadpole-like body shape that results from the distension of the filled buccal cavity. What is different here is the water emerging out of the distended cavity originating from within the mouth rather than from the open ocean, thus contributing very little to the external flows that move about the body (to generate drag). Even in the shape of a tadpole, and while gliding following a lunge, the level of body streamlining is high enough to lead to comparatively lower drag coefficients, i.e., in the range of *C*_*D*_ ~ 0.003–0.005, as with other streamlined animals or solid bodies [30, 37]. Interestingly, rorqual body drag during filtration is quite small in comparison to that sustained *during* a lunge, typically smaller by a factor of 3 [27]. In other words, when taking into account the drag sustained over foraging in addition to filtration, both balaenids and rorquals sustain three-fold increases in drag, in comparison to that of non-feeding travel.

### Large body size is always better for CFF

Increasing cross flow filtering efficiency begins with delaying the onset of filter clogging by reducing the flow speeds across the filtering surface. Speed reduction can be obtained by lowering the lateral pressure gradient, or better, by increasing the area of the filter in relation to that of the inlet [9]. The latter follows not only from BHC modeling, but more generally from mass flow rate conservation within the mouth and baleen. Further, and as mentioned in *Results* (sub-section *Mouth viscous friction drag*), slow intra-baleen flows reduce the drag associated with baleen, thereby enhancing the energy economy of large filtering surfaces.

**Fig 14:**
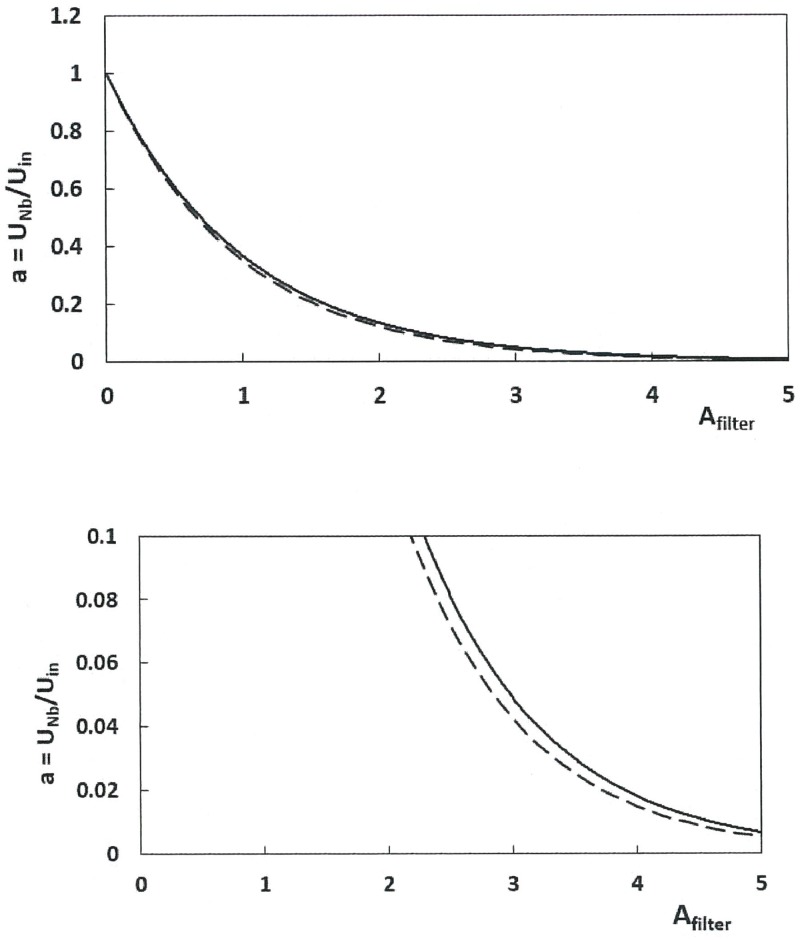
APT flow speeds near the oropharyngeal wall decrease with larger filtering surface area (*A*_*filter*_) relative to mouth area (*A*_*in*_). Calculated from Eq 16. Note that *A*_*filter*_ is equal to the ratio *N*_*b*_ *A*_*IBcanal*_*/A*_*in*_ and thus proportional to the number of baleen plates (*N*_*b*_). Full scale plot (top) versus reduced scale plot (bottom). The continuous and dashed curves correspond to *A*_*IBcanal*_*/A*_*in*_ = 0.02 and 0.20 respectively.

Another (and less obvious) way of increasing efficiency is to reduce sea water contents of the prey/water slurry approaching the oropharyngeal wall, in order to lower the osmotic load of the kidneys, as well as to reduce speed of the nearby APT flows to facilitate swallowing a bolus of accumulated prey. Both feats are accomplished simultaneously since slower APT speeds occur at the end of both racks, again by virtue of mass rate conservation and by having a very large filtering surface in comparison to the mouth inlet area (Fig 4). (The filtering surface areas is 9m^2^ in comparison to mouth area of 1.5m^2^. Here *A*_*filter*_ = 2 *h*_*Ht*_ 300 *d*_*baleen*_, with *h*_*Ht*_ = 1.5m and *d*_*baleen*_ = 0.01m for a 10m whale using 300 baleen plates per rack; see Table 1 and Fig 2; and *A*_*mouth*_ = 2 *D*_*in*_ *h*_*Ht*_, with *D*_*in*_ = 0.5m). As further discussed in *Modeling Details 6* (Eq 21), the ratio *a* of APT flow speeds at the last baleen plate, to that at the rostrum (or *a = u*_*300*_*/U*_*whale*_ in a 300 baleen-per-rack system) can in fact be estimated by *a ~ (1—CA*_*IBfilter*_*/A*_*in*_*)*^*Nb*^ (with *N*_*b*_ = 300). Clearly, the latter (and Fig 14) implies slower oropharyngeal flows with larger filtration area, which in turn supports the idea of large body sizes promoting greater CFF efficiency.

### Final remarks

The BHC model is a simplified description of oral cavity hydrodynamics in balaenid whales. Albeit of low-spatial resolution, this model takes into account the viscous friction at work, particularly through the baleen canals which substantially increases a whale’s wetted surface area. The model relies on several measureable morphometric inputs, along with hydrodynamic data obtained from a flume study of baleen rack [9]. BHC modeling yields a mapping of the pressures and flow speeds, both mediolaterally and anteroposteriorly, and an estimate of the drag generated by the mouth’s viscous friction. So far, the calculated total drag agrees well enough with tag data [18]. Finally, the modeling has highlighted the role of large body size in enhancing filtration efficiency.

With the essential links between body drag and the patterns of intra-oral patterns flows now identified, further study is needed to clarify the role of several factors unmentioned here, preferably using Computational Fluid Dynamics (CFD) simulations of the model cavity. Most useful would be the clarification of the role of the flows entering the two gaps at the mouth’s inlet dorsal to each baleen rack (currently closed), as created by the canted lips and lowered mandibles (compare Figs 2 and 3). The results discussed herein suggest their overall effects on drag as minor; but these could be important enough for the correct determination of the friction coefficients of the IB canals along the anterior portion of the racks, perhaps eliminating altogether the need for their adjustment (“tuning”). Another issue in need of investigation would be the dependence of flow splitting on IB canal-position along the rack. According to the recent flume study [9], the splitting coefficient (*C*) would increase by as much as 25% at locations closer to the inlet.

Although much could be learned from the CFD simulations of flows moving through the rectangular cuboid cavity of Fig 3, the logical next step would be the use of a more realistically-shaped oral cavity, i.e., curved both laterally and dorsoventrally (Fig 1), and incorporating the varying length of baleen along a rack. This is a challenging task that would require not only an accurate characterization of the oral cavity shape in all three dimensions, but also of baleen chord-wise curvature which changes along the span [38]. Curvature may be important not only for understanding the drag production by those hydrofoils, but also the lift that they may also produce (more energy dissipation) and the possible baleen-tip bending and movement that could follow. The latter would help further understand the wear patterns of baleen [39]. Finally, more work will be needed to clarify the role of the tongue’s shape and bulk within the oral apparatus. Per Fig 2, the volume of the tongue is important for the determination of *D*_*in*_ and ultimately for drag control.

### Modeling Details 1. Input data entry sequence

BHC simulations manage information in an order that is opposite to CFD simulations. With the latter, the flows and pressures are calculated first via the numerical solution of the Navier-Stokes equation [26], using input about the morphology and geometry of the body and computational domain. The computation of the drag force follows in a last step, based on the integration of the calculated pressures and velocity gradients along the surface of the object [26]. In the BHC, morphology is also entered first but is followed by the calculation of the pressure *P*_*ext*_ external to the PO using an empirically-suggested flow speed. This enables the calculation of the drag force via Eq 3. Calculation of the internal flow speeds becomes the third step of this process, using the solutions of Tables 5–7. The final step involves adjusting and calculating the values of the friction coefficients which then yield the internal pressures. This last step aims at insuring that the BHC-calculated pressures and dissipated viscous energy in the baleen system are consistent with the prescribed flow speeds and predictions of Eq 3. These essential elements are described here and in the five sections that follow. This section begins with input entry, followed by *Modeling Details 2* which describes the establishment of the constraints applied in the simulations. The calculations of the flows, pressures and drag are then explained next. The last section, *Modeling Details 7*, deals with the generalization of the equations listed in Tables 5–7 –valid for a four-baleen-per-rack case—to one with an arbitrary number of baleen (*N*_*b*_).

The BHC model is simple enough to run in Excel spreadsheet format on any laptop or desktop platform. A simulation begins with the entry of the following basic parameters: the known relevant morphology, namely baleen mean chord (*c*_*baleen*_), (mean) half-height (*h*_*HT*_) and separation (*d*_*baleen*_) (Table 1); the (effective) lateral separation (*D*_*in*_) of baleen from the tongue wall (Fig 2); fluid characteristics such as seawater mass density and kinematic viscosity, along with the sea level atmospheric pressure (Table 2); and whale swimming speed (*U*_*whale*_), as well as that of the flows entering the mouth (*U*_*in*_). (These have been approximated as being the same herein). The number of baleen per rack (*N*_*b*_) is hard-coded in the spreadsheet as a determinant of the number of cells per column. Working with different values of *N*_*b*_ is possible but requires spreadsheet re-configuration.

The value of the empirical flow-splitting coefficient (*C* = 0.30, 0.44 or 0.53 [9]) is calculated next, via the entry of input *a* defined as the ratio *U*_*Nb*_*/U*_*in*_ of the APT flow speed near the posterior-end of the rack (next to oropharynx), over that of the mouth’s entrance. The relationship between input *a* and *C* is given by Eq 21b (below).

Next in the sequence is the input of the mean value of the Posterior Opening width (*w*_*PO*_), calculated from the constraint *w*_*PO*_ = *D*_*in*_/1.15 (Table 4) originating from the specifics of the flows external to the body and moving near and outside the PO. These external flows are at the source of the low pressure *P*_*ext*_ necessary to maintain the internal flows along and across baleen [3, 4]. How the value of *P*_*ext*_ and factor “1.15” are arrived at is further explained in *Modeling Details 2*.

**Fig 15:**
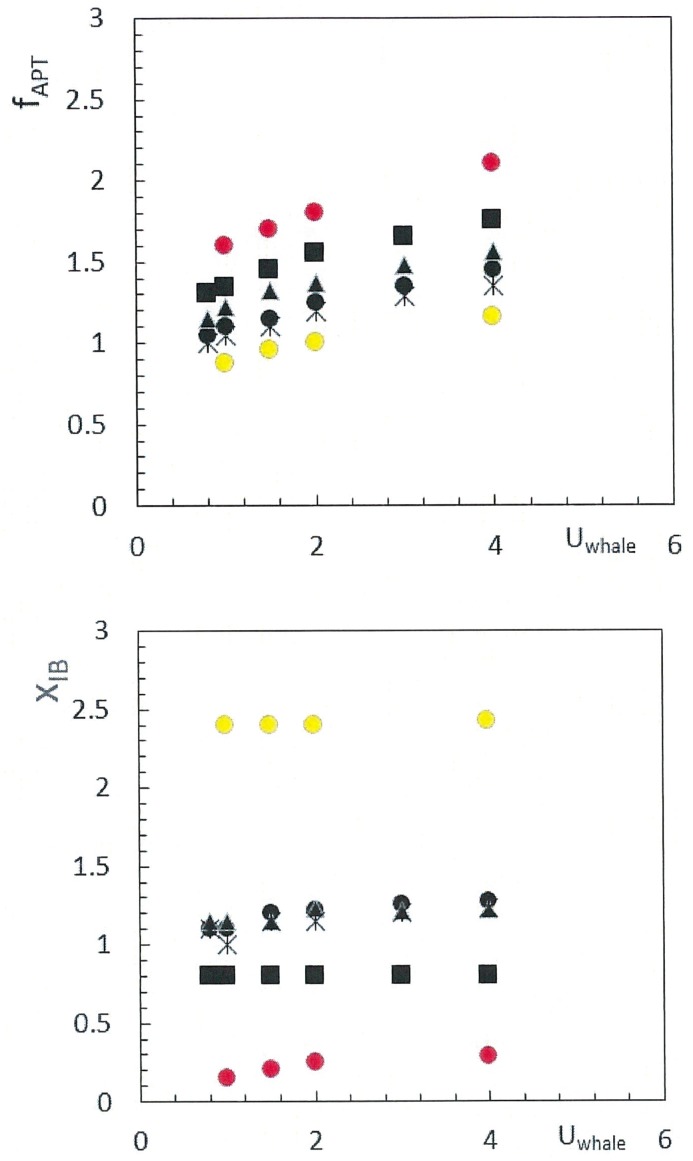
Input values for the coefficients *f*_*APT*_ and *x*_*IB*_ necessary for calculating *k*_*APT*_ and *k*_*IB*_. These inputs are used in Eqs 13 and 15. In both frames the circles correspond to a 10m body size (*D*_*in*_ = 0.50m (black), 0.30m (red) and 0.72m (yellow)); squares to 15m-case A and triangles for 15m-case B; and starburst for 8m. In all of these cases values of *y*_*rostr*_ and *z*_*rostr*_ have been set to *y*_*rostr*_ = *0*.*60* to *0*.*70* and *z*_*rostr*_ = *0*.*0085*.

Two final steps are carried out to finalize the friction coefficient profiles (*k*_*canal*_) in the IB and APT canals (Eqs 13–16). The first consists in adjusting the values of parameter *f*_*APT*_ in Eq 15 to yield, via Eq 10, the internal pressure at the PO (*P*_*PO*_). The latter is usually set at 10–20 Pa higher than the external pressure *P*_*ext*_ to insure a favorable pressure gradient just outside the PO. Representative values are shown in Fig 15. (The variations originate from the acceptable values of *f*_*APT*_ that put the difference *P*_*PO*_*−P*_*ext*_ within this desired 10-to-20Pa interval). The second step adjusts the values of parameters *x*_*IB*_, *y*_*rostr*_, *z*_*rostr*_ (Eq 13), to insure a monotonically decreasing pressure on the labial side of each baleen canal (*P*_*pi*_), i.e., from rostrum to oropharynx (Fig 4). These adjustments are also made so to yield a value of the energy dissipated through all IB canals, and corresponding mouth drag contribution, that is identical to that of the second term in Eq 3. Example values are also shown in Fig 15. Ultimately, both sets of friction coefficient adjustments are necessary to insure consistency with the BHC flow speed solutions (Table 6) which exclude the possibility of backflows into either IB or AP canals.

### Modeling Details 2. Constraints on the flows external to the posterior opening

A filter-feeding balaenid whale deflects a significant mass of seawater around its head even with the mouth open (Fig 1), effectively accelerating this flow in the vicinity of the PO (*U*_*ext*_). Such an effect is also seen with the flows moving over the upper surface of any airfoils, typically yielding velocities that are 15%– 25% higher than in the freestream [40, 41]. These speeds become associated with a zone of low external pressure (*P*_*ext*_) near the PO, which in turns generates the internal pressure gradients of CFF [3, 4]. As such, these external currents interact with the internal flows coming out of the PO (*U*_*PO*_). The values of *U*_*PO*_, *U*_*ext*_, *P*_*ext*_ and *P*_*PO*_ are calculated during the input entry process as follows.

#### Values of *U*_*PO*_ versus *U*_*ext*_

The external flow speed is set to *U*_*ext*_ = *1*.*25 U*_*whale*_ per airfoil aerodynamics [40, 41]. (Using different values of this coefficient has been considered as well, as further discussed below). On the other hand, the internal flow emerging from the PO is also expected to flow at commensurate speeds, that is, *U*_*PO*_ *~ U*_*ext*_. On the other hand, the viscous friction within the oral apparatus is expected to reduce the value of *U*_*PO*_ somewhat, in comparison to what could be obtained in a world free of viscosity [37]. In nozzles, such an effect is measured through the so-called *discharge coefficient*, which in the case of open-ended tubes feature outlet velocities varying in the range of 0.7–0.9 of the theoretical, zero-viscosity velocity [37]. In the case of the gently narrowing tubes that approximate the shape of inflating apex-vented parachutes (early stage), the associated discharge coefficient stand at about 90 to 94% per a recent CFD study [42]. Approximating each half of the oral cavity as one such tube would yield *U*_*PO*_ *~ 0*. *92 U*_*ext*_ ~ *0*.*92* x *1*.*25 U*_*whale*_ = *1*.*15 U*_*whale*_. Most importantly, fixing *U*_*PO*_ and *P*_*PO*_ (*P*_*ext*_) this way determines the essential physics, that is, the level of viscous friction that in turns yields the drag calculated by Eq 3. With *U*_*PO*_ thus assumed, the conservation of the flow mass rate (*w*_*PO*_ *h*_*HT*_ *U*_*PO*_ = *D*_*in*_ *h*_*HT*_ *U*_*in*_) is used to arrive at a constraint on the PO width, namely, *w*_*PO*_ = *D*_*in*_*/1*.*15* (Table 4).

#### Values of *P*_*PO*_ versus *P*_*ext*_

Parameter *P*_*ext*_ is calculated during the data entry process via Bernoulli’s principle,

$$P_{in}+\frac{1}{2}\rho_{w}U_{in}{}^{2}=P_{ext}+\frac{1}{2}\rho_{w}U_{ext}{}^{2}$$ (7)

The pressure *P*_*in*_ is the known hydrostatic pressure at the mouth and at depth (*h*_*HT*_) (also calculated during input entry), *U*_*in*_ the inlet flow speed (= *U*_*whale*_), and *U*_*ext*_ is constrained to *U*_*ext*_ = *1*.*25 U*_*whale*_ as previously discussed. On the other hand, insuring *P*_*PO*_ as greater *P*_*ext*_ by about 10–20 Pa is done through the adjustment of the APT friction coefficient profile (see also *Modeling Details 4*). Knowing the value of *U*_*PO*_, *U*_*ext*_, *P*_*ext*_ and *P*_*PO*_ basically fix the output of Eq 3’s first term. The second term of Eq 3 is also calculated during input data entry, and is used to adjust the values of the IB canal friction coefficient and internal pressures.

The constraints are used at all swim speeds investigated, as well as at all body sizes, values of mouth openings (*D*_*in*_ and *h*_*HT*_) and flow-splitting coefficient (*C*). Changing these constraint slightly, i.e., in accord with the discharge coefficient of 92% and accelerated flows about airfoils, does little in changing the drag. Both cases where *U*_*PO*_*/U*_*whale*_ = *1*.*1* along with *U*_*ext*_*/U*_*whale*_ = *1*.*20*, and *U*_*PO*_*/U*_*whale*_ = *0*.*97* with *U*_*ext*_*/U*_*whale*_ = *1*.*15*, have yielded similar mouth drag values at all body sizes and swim speeds, i.e., within 10–20%, depending on the chosen values of *f*_*APT*_ and *x*_*IB*_.

### Modeling Details 3. Baleen Hydraulic Circuit (BHC) model (four baleen plates)

Flows within oral apparatus are simulated with a low-spatial resolution scheme, illustrated here with a pedagogical 4-baleen example (Fig 3 and Tables 4–6). The more general case of *N*_*b*_ baleen plates is discussed further below (in *Modeling Details 7*) and follows straightforwardly from this example.

The BHC model calculates pressure and flow speeds averaged across pipe/canal sections at the 20 “stations” shown in Fig 3. Because the APT flow loses mass to the baleen canals adjacent to it, a first set of equations is obtained from mass rate conservation, for example as applied to the five-outlet pipe system discussed in [9]:

AinUin=AIBUIB1+AIBUIB2+AIBU+IB3AIBUIB4+AinUout (8)

The specific form used along a four-baleen rack is listed in Table 5. Another set of equations is required to characterize how much of the APT flow at a given station (e.g., station *i*) enters the next IB channel (at station *(i+1)*):

$$U_{p}{}_{\left(i+1\right)}=C\cdot U_{\left(i\right)}$$ (9)

See also Table 5. Generally the *flow-splitting coefficient C* depends on the location of specific IB canals along the rack as a result of the pressure gradients near the canal’s inlet and outlet (see Eq 14 below). But with the intra-baleen canal spacing being small relative to rack length, coefficient *C* is hypothesized as being only weakly dependent on location, and meaningfully obtained from flow tank data [9]. Note that the station next to the oropharynx (i.e., *i = 4* and *pi = p5* in Fig 3) involve different flow equations given their location at the end of the baleen rack.

Solving the BHC equations begins by expressing the IB flow speeds in terms of APT speeds via the merging of Eqs 8 and 9. The resulting equations are used then, station by station, to obtain the APT flow speeds, starting with *U*_*1*_, then *U*_*2*_, and so on. IB flow speeds (*U*_*p1*_, *U*_*p2*_, etc.) are then calculated from the known APT speeds (again via the merged Eqs 8 and 9), to yield Tables 5 and 6. The calculation of the pressure drops then follows via Eq 9 (below) using the now known values for *U*_*i*_, *U*_*pi*_ (and *k*_*canal*_; see Eqs 13 and 14 below), resulting in in the equations listed in Table 7.

It is also straightforward to calculate the flow speeds in the APL canal (*U*_*Li*_) once *U*_*i*_, and *U*_*pi*_ have been computed via the flow mass rate conservation (*w*_*PO*_ *h*_*HT*_ *U*_*Ln*_ = *d*_*baleen*_ *h*_*HT*_ *U*_*Pn*_ *+ w*_*PO*_ *h*_*HT*_ *U*_*Ln+1*_). An example result is shown in Fig 4.

### Modeling Details 4. Pressure drop equations and boundary layer thickness

The pressure profile along the axial length of constant-diameter channels, canals or pipes depends mainly on the size of the boundary layer lining their walls [26, 37] and can be calculated from the following relationship relating the pressure drop at the canal’s ends to flow speed at its inlet [26]:

$$\Delta P\equiv k_{canal}\left(\frac{1}{2}\rho_{w}U_{canal}{}^{2}\right)$$ (10)

Typically, *U*_*canal*_ is the average flow speed in cases where the pipe flow accelerates, or as the flow speed at the inlet where it does not accelerate [26, 37]. The thicker the boundary layer and ensuing energy loss of the flow, the higher *k*_*canal*_ and requisite pressure gradient. Because of the assumed flat wall geometry of each canal, the variation of *k*_*canal*_ along the baleen rack can actually be partly modelled from the theory of boundary layers over flat plates as described below [26]. In a BHC simulation the pressure drops are calculated from Eq 10, with the flow speeds and friction coefficients calculated in previous steps. How values of *k*_*canal*_ are obtained is explained next.

Derivation of the coefficient *k*_*canal*_ applying to the IB canals (≡ *k*_*IB*_) is based on a model of undeveloped pipe flows in which the boundary layer thickness is significantly smaller than the pipe diameter, a result of the larger Reynolds numbers at play (Table 3). The canal flow moving “outside” the boundary layer is seen as laminar and describable by Bernoulli’s principle [26]. (Such a view of Bernoulli flow moving “on top” of the boundary layer is enabled by the concept of the *displacement layer* [26]). Within the boundary layer, on the other hand, the flow is regarded as turbulent (again, because of the Reynolds number) and following the empirical flow velocity profile of along a flat plate [26], which grows over the distance *x* as follows (measured from the inlet):

$$\delta (x)=0.37{\left(\frac{\nu }{U_{in}}\right)}^{1/5}x^{4/5}$$ (11)

With the kinematic viscosity *ν* ranging from 1.05 to 1.45 x10^-6^ m^2^/s, one typically finds small boundary layer thicknesses at the outlet of each IB canals, namely, *δ* ~ 0.003m with the baleen channels located near the rostrum (where *U*_*in*_ = 0.8 m/s), and δ ~ 0.0048m near the oropharynx (where *U*_*in*_ ~ 0.06 m/s). These values are indeed small in comparison with the 0.01m-baleen baleen separation defining the width of IB canal (which are typically 0.07m-long laterally).

With undeveloped flows, the presence of the (displacement) boundary layer growing downstream creates an ever-smaller constriction for the Bernoulli flow to pass through, thereby accelerating it and reducing its ambient pressure [26]. Such gain in speed at the outlet is expressed in terms of the inlet speed via conservation of the mass flow rate through the canal as follows [26]:

Uoutlet=Uinletdbaleendbaleen−28δoutlet (12)

The length *δ*_*outlet*_ corresponds to the boundary layer thickness at the IB canal outlet, as evaluated from Eq 11 (with *x = c*_*baleen*_). The factor 1/8 arises from the calculation being performed with respect to the displacement thickness, a length scale that is smaller than *δ* (Eq 11) by a factor 1/8 [26]. The pressure drop along both ends of a given IB canal *(≡ ΔP*_*i*_*)* can be determined by merging Eqs 10 and 12 with *U*_*inlet*_ = *U*_*pi*_ and Bernoulli’s equation (*ΔP*_*i*_ = *P*_*i*_*—P*_*pi*_ = *½ ρ*_*w*_ *[(U*_*outlet*_*)*^*2*^ *–(U*_*inlet*_*)*^*2*^*]*), to yield *k*_*IB*_*(i) = (d*_*baleen*_ */(d*_*baleen*_*−δ(i)/4))*^*2*^*–1* [26]. Here the index *i* represents the baleen canal being considered along each rack. (The distance to the rostrum is given by *i d*_*baleen*_).

To this value of *k*_*IB*_ one must add (first) the friction losses attributed to the two bending paths a given IB current has to make to ultimately reach the PO, namely, from APT to IB, and then from IB to APL. In hydraulics, these are attributed to so-called *secondary* flows which move fluid off-axis within each bend [26, 35, 36]. Given the associated values of the Reynolds numbers (~ 10^3^; Table 3), such losses significantly rises the value of *k*_*IB*_, namely up to 1.0 to 3.0 per units of *ΔP/0*.*5ρU*^*2*^ [35, 36]. The IB canal friction coefficient that results is written as follows:

kcanal≡kIB(i)=xIBbend{1−yrostre−zrostri}+[(dbaleendbaleen−14δIB(i))2−1] (13)

$$\delta_{IB}(i)=0.37{\left(\frac{\nu }{U_{pi}}\right)}^{1/5}c_{baleen}{}^{4/5}$$ (14)

The contribution of the two bends is represented by coefficient *x*_*IB*_ which is set in the range of 0.5 to 2.5 (Fig 15), following a comparison of the second term in Eq 3 with a direct calculation of energy dissipation and drag produced (Eq 20). Such values turn out as quite typical in the microfluidics of bends [35] and hydraulics of small diameter pipes [36].

**Fig 16:**
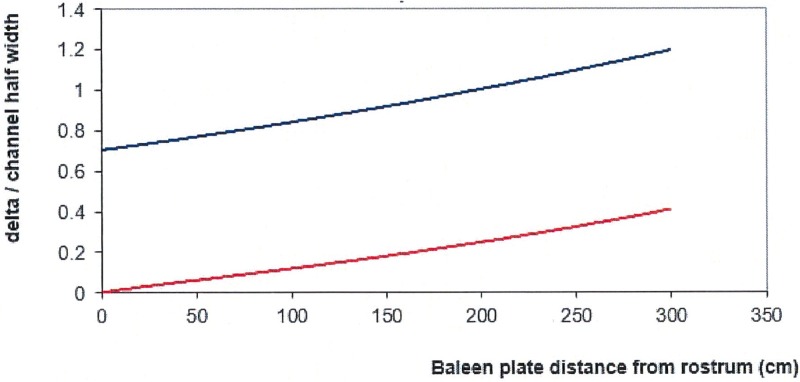
BHC boundary layer thickness ratio at the end of each IB canal (top), and along the APT canal (bottom). The ratio is defined as δ/(0.5*d_baleen_), with δ calculated from Eqs 14 and 18. The data shown corresponds to a 10m whale swimming at *U*_*whale*_ = 1.0m/s. The rest of the input parameters are the same as those of Fig 4.

The function appearing within the curly brackets in Eq 13 is an *ad hoc* addition designed to enable a monotonically-decreasing pressure along the APL canal (*P*_*pi*_), typically in the anterior third portion of each rack (Fig 4). The value of *y*_*rostr*_ and *z*_*rostr*_ deemed necessary to achieve this are in the range *y*_*rostr*_ = *0*.*60–0*.*70* and *z*_*rostr*_ = *0*.*0085*. Fig 5 shows a typical IB friction coefficient profile along the baleen rack of a 10m whale, and Fig 16 shows a typical boundary layer thickness (Eq 14). The latter indeed turns out smaller than half the canal width as expected (undeveloped flows) and over most of a rack. Where the boundary layers meet, the corresponding *k*_*IB*_-values are larger—but by only a factor 1.05x - than the values found in pipe systems supporting fully-developed and turbulent flows at the same Reynolds number (~ 10^3^–10^4^) [26].

Modeling the friction coefficient in the APT canal proceeds similarly. With this canal formed by the tongue and baleen rack itself (Fig 1), there is only one continuous wall which in turns supports only one continuous boundary layer. Further, loss of fluid mass through the adjacent IB canals results in decreasing APT flow speeds per mass rate conservation [9] (Fig 4). Modeling APT pressure drops at each station again begins with using Eq 10 but now applied to “pipe” sections connected in series, each of length spanning baleen separation *d*_*baleen*_. These pipes are again assumed to support undeveloped flows, with a Bernoulli flow accelerating atop of the displacement boundary layer growing along the lingual wall. Per the mass rate conservation one has *U*_*inlet*_ *h*_*HT*_ *D*_*in*_ = *U*_*outlet*_ *h*_*HT*_ *(D*_*in*_*—δ*_*(outlet)*_*/8)* (with *U*_*inlet*_ = *U*_*i*_), which in turns yields:

kcanal≡kAPT(i)=fAPT{(DinDin−(δAPT(i)8))2−1} (15)

δAPT(i)=0.37(νUi)15(idbaleen)45 (16)

The effect of adjacent IB canal on overall friction is accounted through *f*_*APT*_, which is tuned during input-entry so to lower *P*_*Nb*_ and *P*_*pNb*_ to values slightly above *P*_*ext*_ (Fig 4; and *Modeling Details 2*), resulting in the values shown in Fig 15. An example of boundary layer thickness along the APT canal calculated via Eq 16 is also shown in Fig 16.

The algorithm based on Eq 10 yields pressure profiles along the APT and APL canal which are continuous within numerical discretization variability (Fig 4). It follows that they yield the same pressure difference between any two points in the cavity, via integration of pressure-increments along arbitrary paths in the APT and/or APL canals starting and ending at those same two points. For example, calculating the pressure difference (*P*_*6*_ *–P*_*p6*_) directly from Eqs 30 and 32 yields a value of 51.94Pa, in comparison to 51.78Pa obtained from integrating the pressure decrement (*P*_*i+1*_ *–P*_*i*_) in the APT canal from site *i* = 6 and to site *i* = 200, plus the difference *P*_*200*_ *–P*_*p200*_ (following a path through IB canal #200), and finally adding the integration of the decrements (-*P*_*pi+1*_ *+ P*_*pi*_) in the APL canal from *i* = 200 to *i* = 6. The 0.3% difference that results is typical of the many paths that have been tested.

### Modeling Details 5. Viscous friction drag of the oral apparatus (Eq 3)

The drag contributed by the oral apparatus is obtained by considering the rate of energy dissipated by the flows moving through it. This follows from the fact that with bodies traveling through a viscous fluid at constant speed and under the action of a single external and forward-directed force (thrust), the work done by the latter turns out as identical to the energy (*E*_*friction*_) dissipated by the viscous friction sustained by, and turbulence imparted to the fluid. With drag being equal to thrust, and over travel time scale *T*, one therefore has *F*_*D*_ = *E*_*friction*_*/(U*_*whale*_*T)*. (Note that this approach would not be correct if the body were to accelerate, since drag is no longer equal to thrust, and more importantly, since drag generation would then consume more energy beyond viscous friction and turbulence, i.e., in the form of the extra kinetic energy gained by the accelerating fluid. This latter effect leads to the so-called *acceleration reaction* or *added mass drag* [10]).

In what follows, scale *T* will be chosen as the time needed for the whale to swim a distance equal to one body length. Eq 3 is derived by estimating the rate of energy dissipation (*E*_*friction*_*/T*) inside the mouth, here seen as a juxtaposed pair of boxes each featuring laterally-offset inlets and outlets, and carrying a rack of hydrofoils (baleen) of known drag characteristics. In this model, the energy dissipated anteroposteriorly (AP) is calculated independently from that dissipated by the lateral (IB) flows moving past the hydrofoils.

Generally, the rate of viscous dissipation arises from a turbulence component, dominant at high Reynolds numbers, superposed to a frictional component that dominates at low Reynolds number. The effects of the former can be isolated following an argument based on Reynolds-averaging [43], wherein the flow field is decomposed into a time-averaged flow speed *U* and a time-dependent fluctuating component *u* for which the time-averaged values are $\bar{u}=0$ and $\bar{u^{2}}\neq 0$. With a 2-dimensional velocity field (i.e., *longitudinal* and *lateral*) going through any cavity geometry, Reynold’s averaging of the energy density (J/m^3^) yields:

$$\begin{array}{l} P_{in}+\frac{1}{2}\rho_{w}{\left(U_{long}{}^{2}\right)}_{in}+\frac{1}{2}\rho_{w}{\left(U_{lat}{}^{2}\right)}_{in}=\\ P_{PO}+\frac{1}{2}\rho_{w}{\left(U_{long}{}^{2}\right)}_{PO}+\frac{1}{2}\rho_{w}\bar{{\left(u_{long}{}^{2}\right)}_{PO}}+\\ \frac{1}{2}\rho_{w}{\left(U_{lat}{}^{2}\right)}_{PO}+\frac{1}{2}\rho_{w}\bar{{\left(u_{lat}{}^{2}\right)}_{PO}}+Q_{friction} \end{array}$$ (17a)

Here the flows entering the cavity are assumed as purely laminar and characterized by a total energy given by Bernoulli’s equation. The energy loss term *Q*_*friction*_ represents the effects of the frictional component mentioned above, as accumulated by the fluid particles when they reach the PO. All of the variables above are also understood as averages over the relevant cross section areas at the inlet and outlet/PO. Associating the time averages of *u*_*long*_ and *u*_*lat*_, and the friction loss *Q*_*friction*_ with the dissipated energy densities *e*_*friction*_^*long*^ and *e*_*friction*_^*lat*^ one has

$$\begin{array}{l} e_{friction}{}^{long}+e_{friction}{}^{lat}=\left(P_{in}-P_{PO}\right)+\frac{1}{2}\rho_{w}{\left(U_{long}{}^{2}\right)}_{in}-\frac{1}{2}\rho_{w}{\left(U_{long}{}^{2}\right)}_{PO}\\ +\frac{1}{2}\rho_{w}{\left(U_{lat}{}^{2}\right)}_{in}-\frac{1}{2}\rho_{w}{\left(U_{lat}{}^{2}\right)}_{PO} \end{array}$$ (17b)

The more friction and turbulence losses there are, the larger the lateral velocity and inlet pressure terms on the right-hand-side of Eq 17b. But modifications are necessary since the BHC neglects altogether the existence of the lateral flows (*U*_*lat*_) at both the inlet and PO, in effect eliminating the last two terms in Eq 17b. The BHC also use inlet speed and pressure (*U*_*in*_ and *P*_*in*_) that are set equal to those of the freestream (*U*_*whale*_ and *P*_*atm*_ *+ ρ*_*w*_*gh*_*HT*_), which precludes any increases in *P*_*in*_ that would arise from internal energy dissipation.

The first set of modifications involves using the first three terms of Eq 17b, but with the *U*_*long*_ and pressure terms calculated in terms of the parameters *U*_*whale*_, *U*_*PO*_, *U*_*ext*_, *P*_*ext*_ and *P*_*PO*_ described in *Modeling Details 2*. This would approximate the viscous dissipation of the anteroposterior flows moving along smooth walls (via *U*_*PO*_). The second modification adds a new term to Eq 17b, to accounts for the losses incurred in the lateral direction, by associating the viscous energy dissipated by the through-baleen flows to that of flows moving through a rack of hydrofoils of known drag characteristics. This is accounted as follows.

With each baleen plate of known mean span (*h*_*HT*_) and chord (*c*_*baleen*_), and drag coefficient (*C*_*D*_^*baleen*^), the rate of energy dissipation by a system of two racks supporting 300 plates each is readily obtained from the drag generated as *2 x 300 x (½ ρ*_*w*_ *C*_*D*_^*baleen*^ *h*_*HT*_ *c*_*baleen*_ *V*_*lat*_^*2*^*)V*_*lat*_. Here the drag coefficient has been set to *C*_*D*_^*baleen*^ = 0.20/0.8 = 0.25, with the factor 0.8 used to account for the solid blockage effects created by the presence of the two baleen plates next to any given plate [44]. (In effect each hydrofoil has to be seen as being in a water tunnel of sorts, created by the presence of its neighbors). The factor 0.20 is a drag coefficient value measured in a wind tunnel at about -20° angle of attack at low Reynolds numbers (~ 10^4^), on models representative the kinked airfoils used by dragonflies [45, 46]. Using kinked foils follows from the baleen plates having double camber (i.e., concave at the leading edge and convex at the trailing edge) over a good portion of its span (*h*_*HT*_) [38]. The wing models studied in [45] are high-aspect ratio as well, spanning the entire test section (just as with baleen). On the other hand, using data-points at large angles of attack attempts at representing the effects of the fluid curving along bends to enter and exit the IB canals. Finally, the factor *V*_*lat*_ is the lateral flow through baleen (*U*_*pi*_), averaged along each rack in the BHC. Note that *V*_*lat*_ can also be calculated with 5–10% accuracy from the following formula, which is based on the mass flow rate going through the baleen/hydrofoil rack matching the mass flow rate thought the mouth’s inlet:

Vlat=[21+UinUPO]12(Din+wPO)LmouthUin (18)

Parameter *L*_*mouth*_ corresponds to the length of the oral apparatus, and factor ½ (*D*_*in*_ *+ w*_*PO*_) serves as a travel distance scale for the fluid moving laterally within the cavity. The latter is adjusted by the quotient appearing in between square brackets to ensure conservation of the mass flow rate everywhere. Within the assumptions of the BHC, this length scale and *V*_*lat*_ basically grow with mouth inlet size as *D*_*in*_*/L*_*mouth*_ (in accord with the solution shown in Table 6).

The rate of dissipated energy for the entire cavity is thus obtained by adding to the energy dissipated longitudinally (in the right-hand-side of Eq 17b but without the *U*_*lat*_-terms) the energy lost thought the lateral motions through the hydrofoil racks. Multiplying those energy densities by the volumetric rate *D*_*in*_*h*_*HT*_*U*_*in*_ yields the following drag force generated by open mouth:

FDmouth=2DinhHTUinUwhale{12ρw(Uwhale2−UPO2)+(Pin−PPO)+12ρwCDbaleencbaleenLmouthdbaleenDin(21+Uin/UPO)3[12(Din+wPO)Lmouth]3Uin2} (19)

The factor “2” multiplying the large curly brackets adds the effects due to both baleen racks; and setting *U*_*in*_ as equal to *U*_*whale*_ leads to Eq 3.

Calculating the dissipated energy incurred by the lateral flows though baleen/hydrofoils also yields an expression of the body drag contributed by the dissipated energy in each of the *2N*_*b*_ canals (= *k*_*IB*_ *½ ρ*_*w*_ *U*_*pi*_^*2*^), namely

$$F_{D}^{mouth}|{}_{IB}=2\frac{1}{U_{whale}}d_{baleen}h_{HT}\sum_{i}^{Nb}k_{IB}\frac{1}{2}\rho_{w}U_{Pi}{}^{3}$$ (20)

This result follows from using Eq 17b in each IB canal, associating “in” with the inlet and “PO” with the outlet, setting the “lat” speeds to zero, and having the same “long” speeds at both inlet and outlet. From this one obtains *e*_*friction*_^*long*^ = *P*_*in*_*−P*_*PO*_, which along with Eqs 10, 13 and 14 yields the energy density (J/m^3^). Multiplying the latter by volumetric rate *d*_*baleen*_ *h*_*HT*_ *U*_*pi*_ gives the rate of energy dissipation (Watts) in a canal. Summing over all baleen canals and multiplying by the time T spent by the whale to travel a distance equal to its body length (*L*_*body*_) yields the work (*F*_*D*_^*mouth*^ *|*_*IB*_ *L*_*body*_) needed from the whale to replace the energy dissipated in the IB canals and ensuing mouth drag. Eq 20 is another important element of the BHC as it allows, via the comparison with the second term of Eqs 3 or 19, the determination of the final value of *k*_*IB*_ via the tuning of *x*_*IB*_.

### Modeling Details 6. APT flow speeds near the oropharynx and relation to body size

The AP speed at the end of the APT canal is interesting as it corresponds to the speed of the ready-to-eat slurry near the oropharyngeal wall. One can calculate it’s ratio to mouth inlet flow speed as follows (in a 4-baleen system; see Table 6):

$$a\equiv \frac{U_{5}}{U_{in}}={\left(1-\frac{A_{IBcanal}}{A_{in}}C\right)}^{4}$$ (21a)

Cross section areas *A*_IBcanal_ and *A*_*in*_ are those of the IB and APT canals respectively (Fig 2 and Table 4) (Table 4). In the case of a more general *N*_*b*_ = *N-1* baleen system this ratio would be calculated as (see *Modeling Details 7*):

$$a=\frac{U_{N}}{U_{in}}={\left(1-\frac{A_{IBcanal}}{A_{in}}C\right)}^{N-1}$$ (21b)

In the limit where the number of baleen plates (and N) becomes very large, the value of *a* converges to a near-zero value since the ratio *C A*_*IBcanal*_*/A*_*in*_ is less than unity. Note also that this function is also very close numerically to the exponential *a ~ exp(-N*_*b*_ *A*_*baleen*_*/A*_*in*_*)* in the limit of large baleen plate numbers.

### Modeling Details 7. BHC modeling for N_b_ baleen (per rack)

Generalizing the BHC approach to an arbitrary number *N*_*b*_ of baleen per rack (= *N-1*) is straightforward once the 4-baleen case is understood. One begins with the mass rate equation which insures that, over a given time interval, the fluid mass entering a selected APT canal (Fig 3) is equal to the fluid mass exiting through both of the canal’s IB channel and APT outlet:

$$\begin{array}{l} A_{in}U_{in}=A_{in}U_{1}+A_{IBchannel}U_{p1}\\ A_{in}U_{1}=A_{in}U_{2}+A_{IBchannel}U_{p2}\\ A_{in}U_{2}=A_{in}U_{3}+A_{IBchannel}U_{p3}\\ \vdots \\ A_{in}U_{n}=A_{in}U_{n+1}+A_{IBchannel}U_{pn+1}\\ \vdots \\ A_{in}U_{N-2}=A_{in}U_{N-1}+A_{IBchannel}U_{pN-1}\\ A_{in}U_{N-1}=A_{in}U_{N} \end{array}$$ (22)

Again both cross section areas *A*_*IBchannel*_ and *A*_*in*_ known (Table 4). Note the equation for the last pipe section where there is no APT channel to discharge into, per Fig 3 (assuming a closed oropharynx).

Eq 22 ensure that the mass rate entering the tongue-baleen gap canal (*A*_*in*_*U*_*in*_) is identical to the flow rate leaving each posterior opening (PO):

$$A_{in}U_{in}=A_{PO}U_{PO}$$ (23)

This can be shown by noting first that the flow speeds (*U*_*Li*_) in the baleen-lip canal (APL canal) arise from the merging of the flows exiting each IB canal, namely,

$$A_{PO}U_{Li}=\sum_{j=1}^{i}A_{IBchannel}U_{pj}$$ (24)

and from the flow mass rate through the PO is given by

$$A_{PO}U_{PO}=A_{in}U_{N}+A_{PO}U_{LN-1}$$ (25)

Note that the flows *U*_*Li*_ defined above exclude the flow *U*_*L0*_ that enters the APL canal at the rostrum, hence the extra term on both sides of the mass conservation equation above.

The next series of equations tracks the flow splitting:

$$\begin{array}{l} U_{p1}=CU_{in}\\ U_{p2}=CU_{1}\\ U_{p3}=CU_{2}\\ \vdots \\ U_{pn}=CU_{n-1}\\ \vdots \\ U_{pN-2}=CU_{N-3}\\ U_{pN-1}=CU_{N-2}\\ A_{baleen}U_{pN}=A_{in}U_{N-1} \end{array}$$ (26)

As discussed in the text, the value of the flow splitting coefficient *C* is obtained from flow tank data [9] and is assumed to be the same along the full length of each baleen rack.

Merging the above equations and solving for flow speeds proceeds hence, beginning at station *n = 1*, then solving at station 2, and so on. Along the sections of the APT canal the solution is as follows:

$$\begin{array}{l} U_{n}=U_{in}{\left(1-\frac{A_{IBchannel}}{A_{in}}C\right)}^{n}1\leq n\leq \left(N-1\right)\\ U_{N}=U_{N-1}=U_{in}{\left(1-\frac{A_{IBchannel}}{A_{in}}C\right)}^{N-1} \end{array}$$ (27)

On the other hand, each IB channel features a solution of the form:

$$\begin{array}{l} U_{pn}=U_{in}C{\left(1-\frac{A_{IBchannel}}{A_{in}}C\right)}^{n-1}1\leq n\leq \left(N-1\right)\\ U_{pN}=\frac{A_{in}}{A_{IBchannel}}U_{N}=\frac{A_{in}}{A_{IBchannel}}U_{N-1} \end{array}$$ (28)

The flow speeds in the APL channel are obtained by using Eqs 27 and 28 along with mass conservation enforced in this canal (Fig 3):

$$A_{PO}U_{j-1}+A_{IBchannel}U_{pj}=A_{PO}U_{j}$$ (29)

With the flow speeds known everywhere in the buccal cavity, one then calculates the values of the friction coefficients *k*_*IB*_ and *k*_*APT*_ from Eqs 13–16. The corresponding pressure drops then follow via Eq 10:

$$P_{n-1}-P_{n}=k_{APT}(n)\frac{1}{2}\rho_{w}U_{n}{}^{2}1\leq n\leq (N-1)$$ (30)

$$P_{n}-P_{pn}=k_{IB}(n)\frac{1}{2}\rho_{w}U_{pn}{}^{2}1\leq n\leq (N-1)$$ (31)

$$P_{N}-P_{pN}=0$$ (32)

Once the flow speeds and pressure drops in both APT and IB canals are computed, the friction coefficients *k*_*APL*_ in the APL canal is reconstructed using the associated Darcy-Weisbach equation (Eq 10):

kAPL(n)=Ppn−1−Ppn12ρwULn21≤n≤(N−1) (33)

## Abbreviations

*A_in_*: Surface area of each APT canal (Fig 2) (Table 4)
*A_IBchannel_*: Surface area of each intra-baleen canal (or channel) (Table 4)
*A_PO_*: Surface area of the Posterior Opening (Fig 2) (Table 4)
*c_baleen_*: Average chord of a baleen plate
*C*: Flow-splitting coefficient (also a measure of baleen obstruction state)
*C_D_^baleen^*: Drag coefficient of a baleen plate/hydrofoil
*C_D_^closed mouth^*: Drag coefficient corresponding to *F*_*D*_^*total*^ (with closed mouth)
*C_D_^mouth^*: Drag coefficient corresponding to open mouth drag *F*_*D*_^*mouth*^ (Eq 3)
*C_D_^total^*: Drag coefficient corresponding to total body drag *F*_*D*_^*total*^ (with open mouth)
**d*_*IB*_ ≡ *d*_*baleen*_*: Baleen plate spacing along each rack
*D_in_*: Width of an (idealized) APT canal (Fig 2)
*E_friction_*: Energy dissipated through friction and turbulence by the seawater moving within the oral cavity
*e_frict_^long^, e_frict_^lat^*: Energy dissipated longitudinally and laterally by the seawater moving within the oral cavity (Eq 17b)
*f_APT_*: Friction coefficient parameter for APT canal segments (Eq 15)
*F_D_^body^*: Drag force generated by the external flows moving along the body
*F_D_^mouth^*: Drag force generated by the internal flows moving through the oral apparatus
*F_D_^total^*: Total drag force
*h_HT_*: Average height of the baleen plates along each rack (Fig 2)
*k_canal_*: Friction coefficient
*k_APL_*: Friction coefficient for APL canal segments
*k_APT_*: Friction coefficient for APT canal segments
*k_IB_*: Friction coefficient for an IB canal
*L_body_*: Body length
*L_mouth_*: Length of the oral apparatus; equal to *N*_*b*_ *d*_*baleen*_
*M_c_*: Body mass
*N_b_*: Number of baleen plates on one rack
*P_ext_*: Pressure outside and near the Posterior Opening
*P_i_*: Pressure in the APT canal at site *i* (Fig 3)
*P_in_*: Pressure at the mouth’s entrance
*P_pi_*: Pressure in the APL canal at site *i* (Fig 3)
*P_PO_*: Pressure at the Posterior Opening (PO)
*Re*: Reynolds number (= *speed x length/ν*)
*S_wetted_*: Wetted surface area of the body (closed mouth)
*U_ext_*: Speed of seawater flow external to the body and near the PO
*U_in_*: Flow speed entering the mouth (sub-rostral)
*U_i_*: APT flow speed at station *i*
*U_pi_*: Inlet intra-baleen flow speed; at station *i*
*U_Li_*: APL flow speed at station *i*
*U_whale_*: Travel speed of a whale during foraging
*x_IB_*: IB friction coefficient parameter (Eq 13)
*y_rostr_*: IB friction coefficient parameter (Eq 13)
*z_rostr_*: IB friction coefficient parameter (Eq 13)
*δ(x)*: boundary layer thickness a distance *x* along canal wall
*δ_IB_*: boundary layer thickness at the outlet of an IB canal
*δ_APT_*: boundary layer thickness at the outlet of an APT canal segment
*γ*: Drag correction factor due to depth
*ν*: Kinematic viscosity of sea water
*ρ_w_*: Density of seawater
AP: Anteroposterior
APT: Anteroposterior, lingual side
APL: Anteroposterior, labial side
BHC: Baleen Hydraulic Circuit
CFF: Cross-flow filtration
IB: Intra-baleen
PO: Posterior Opening

## References

[1] Werth AJ. 2000 Marine Mammals In: Schwenk K, editor. *Feeding*: *Form*, *Function and Evolution in Tetrapod Vertebrates*. New York: Academic Press pp. 475–514.

[2] Werth AJ. How do mysticetes remove prey trapped in baleen? Bull MCZ. 2001; 156:189–203.

[3] Werth AJ. Models of hydrodynamic flow in the bowhead whale filter feeding apparatus. J Exp Biol. 2004; 207: 3569–3580. 10.1242/jeb.01202 15339953

[4] Werth AJ. Hydrodynamic and sensory factors governing response of copepods to simulated predation by baleen whales. Int J Ecol. 2012;

[5] Werth AJ. Flow-dependent porosity and other biomechanical properties of mysticete baleen. J Exp Biol. 2013; 216:1152–1159. 10.1242/jeb.078931 23487267

[6] Rubenstein DI, Koehl MAR. The mechanisms of filter feeding: some theoretical considerations. Am Nat 1977; 111: 981–994.

[7] Sanderson SL, Wassersug R. Suspension-feeding vertebrates. Sci Am. 1990; 262: 96–101.

[8] Sanderson SL, Wassersug R. 1993 Convergent and alternative designs for vertebrate suspension feeding In: Hanken J, Hall BK, editors. *The Skull Vol*. *3*: *Functional and Evolutionary Mechanisms*. Chicago: Univ. of Chicago Press pp. 37–112.

[9] Werth AJ and Potvin J. Baleen Hydrodynamics and Morphology of Cross-Flow Filtration in Balaenid Whale Suspension Feeding. PLoS ONE. 2016 11(2): e0150106 10.1371/journal.pone.0150106 26918630 PMC4769178

[10] Denny M. Air and Water. Princeton: Princeton University Press 1993.

[11] Goldbogen JA, Cade DE, Calambokidis J, Friedlaender AS, Potvin J, Segre PS, et al How Baleen Whales Feed: The Biomechanics of Engulfment and Filtration. Annual Review of Marine Science. 2017; 9: 367–386. 10.1146/annurev-marine-122414-033905 27620830

[12] Goldbogen JA. The Ultimate Mouthful: Lunge Feeding in Rorqual Whales. American Scientist. 2010; 98: 124–131.

[13] Goldbogen JA, Calambokidis J, Kroll DA, McKenna MF, Oleson E, Potvin J, et al Scaling lunge feeding performance in rorqual whales: mass-specific energy expenditure increases with body size and progressively limits diving capacity. Funct Ecol. 2012; 26: 216–226.

[14] Orton LS, Brodie PF. Engulfing Mechanics of Fin Whales. Canadian Journal of Zoology. 1987; 65(12): 2898–2907.

[15] Shadwick RE, Goldbogen JA, Potvin J, Pyenson ND, Vogl AW. Novel muscle and connective tissue design enables high extensibility and controls engulfment volume in lunge-feeding rorqual whales. The Journal of Experimental Biology 2013 216(14): 2691–2701. 23580724 10.1242/jeb.081752

[16] Fontaine P-H. Whales and Seals Biology and Ecology. Atglen PA: Schiffer Publishing Ltd 2007.

[17] Berthier J, Silberzan P. Microfluidics for Biotechnology. Norwood, MA: Artech House 2009.

[18] Nousek McGregor AE. The cost of locomotion in North Atlantic Right Whales Eubalaena glacialis. Ph.D. Thesis. Duke University. 2010.

[19] Hain JHW, Hamp JD, McKenney SA, Albert JA, Kenney RD. Swim Speed, Behavior, and Movement of North Atlantic Right Whales (Eubalaena glacialis) in Coastal Waters of Northeastern Florida, USA. PLoS ONE. 2013; 8(1): e54340 10.1371/journal.pone.0054340 23326603 PMC3542314

[20] Baumgartner MF, Mate BR. Summertime foraging ecology of North Atlantic right whales. Mar. Ecol. Prog Ser 2003; 264: 123–135.

[21] Kenney RD, Hyman MA, Owen RE, Scott GP, Winn HE. Estimation of prey densities required by western North Atlantic right whales. Mar Mamm Sci. 1986; 2(1): 1–13.

[22] Simon M, Johnson M, Tyack P, Madsen PT. (). Behavior and kinematics of continuous ram filtration in bowhead whales (Balaena mysticetus). Proc R Soc B. 2009; 276: 3819–3828. 10.1098/rspb.2009.1135 19692400 PMC2817290

[23] Vogel S. Comparative Biomechanics: Life’s Physical World. 2^nd^ edition Princeton: Princeton University Press 2003.

[24] Riedeberger D, Rist U. 2012 Numerical simulation of laminar-turbulent transition on a dolphin using the γ-Re model In *High Performance Computing in Science and Engineering* '11 (ed. Nagel W. E., Kröner D. B. and Resch M.). Berlin: Springer pp. 379–391.

[25] Paig-Tran EWM, Kleinteich T, Summers AP. The filter pads and filtration mechanism of the devil rays: variation at macro and microscopic scales. J Morph. 2013; 274(9): 1026–1043. 10.1002/jmor.20160 23686500

[26] Fox RW, McDonald AT. Introduction to Fluid Mechanics, 2^nd^ Edition, New York NY: John Wiley and Sons 1978.

[27] Potvin J, Goldbogen JA, Shadwick RE. Metabolic expenditures of lunge feeding rorquals across scale: implications for the evolution of filter feeding and the limits to maximum body size. PLoS ONE 2012; 7(9): e44854 10.1371/journal.pone.0044854 23024769 PMC3443106

[28] Goldbogen JA, Potvin J, Fish FE. Hydrodynamics In *Marine Mammal Physiology*: *Requisites for Ocean Living*. Editors: Michael Castellini and Jo-Ann Mellish. pp 3–28. CRC Press 2015.

[29] Fish FE. Comparative kinematics and hydrodynamics of odontocete cetaceans: Morphological and ecological correlates with swimming performance. Journal of Experimental Biology. 1998; 201(20): 2867–2877. 9866875

[30] Kooyman GL. Diverse Divers. New York: Springer-Verlag 1989.

[31] van der Hoop JM, Corkeron P, Kenney J, Landry S, Morin D, Smith J, et al Drag from fishing gear entangling North Atlantic right whales. Marine Mammal Science. 2015.

[32] van der Hoop JM, Moore MJ, Fahlman A, Bocconcelli A, George C, Jackson K, et al Behavioral impacts of disentanglement of a right whale under sedation and the energetic cost of entanglement. Marine Mammal Science. 2013.

[33] Lockyer C. Body weights of some species of large whales. Journal du Conseil International pour l’Exploration de la Mer. 1976; 36: 259–273.

[34] Hertel H. Structure, Form, Movement. New York: Reinhold 1966.

[35] Lam Trung Nguyen LT. Contraction/Expansion Effects in 90° Miter Bends in Rectangular Xurographic Microchannels. MS Thesis, University of Utah. 2011.

[36] Pipe Friction Manual. New York, NY: Hydraulic Institute 1954.

[37] Blevins RD. Applied Fluid Dynamics Handbook. Malabar, FL: Krieger Publishing, 1992.

[38] Lambertsen RH, Rasmussen KJ, Lancaster WC, Hintz JR. Functional Morphology of the Mouth of the Bowhead Whale and Its Implications for Conservation. J. Mamm. 2005 86(2): 342–352.

[39] Werth AJ, Straley JM, Shadwick RE. Baleen Wear Reveals Intraoral Water Flow Patterns of Mysticete Filter Feeding. J Morphol. 2016; 277(4): 453–71. 10.1002/jmor.20510 26825852

[40] Eppler R. Airfoil Design and Data. Heidelberg: Springer-Verlag 1990.

[41] Abbott IH, von Doenhoff AE. Theory of Wing Sections: Including a Summary of Airfoil Data. New York: Dover Publications 1959

[42] Potvin J, Kavanaugh J, McQuilling M. Geometric Porosity, as a Dynamically Driven Process: Inflation versus Steady Descent. Paper AIAA-2015-2182. Presented at the 23th AIAA Aerodynamic Decelerator Systems Technology Conference and Seminar, Daytona Beach, FL, March 30—April 2, 2015.

[43] Wilcox DC. Turbulence Modeling for CFD. 3^rd^ Edition La Canada CA: DCW Industries 2006.

[44] Rae WH, Pope A. Low-speed wind tunnel testing. New York: Wiley Interscience 1984.

[45] Kessel AB. Aerodynamic Characteristics of Dragonfly Wing Sections Compared with Technical Aerofoils. J Exp Biol. 2000; 203: 3125–3135. 11003823 10.1242/jeb.203.20.3125

[46] Shyy W, Lian Y, Tang J, Viieru D, Liu H. Aerodynamics of Low Reynolds Number Flyers. Cambridge: Cambridge University Press 2008.

